# Shear‐Activated von Willebrand Factor Captures Extracellular Vesicles to Promote Platelet Activation and Metastasis

**DOI:** 10.1002/jev2.70349

**Published:** 2026-08-09

**Authors:** Yuanyuan Wang, Xiaobo Liu, Pascal Nakielski, Katrin Nekipelov, Amanda Salviano‐Silva, Alper Topuz, Alexander T. Bauer, Julian Kött, Jannis Akrivakis, Santra Brenna, Tomasz Downar, Neus Feliu, Gerd Bendas, Berta Puig, Stefan W. Schneider, Dmitry A. Fedosov, Christian Gorzelanny

**Affiliations:** ^1^ Department of Dermatology University Medical Center Hamburg‐Eppendorf Hamburg Germany; ^2^ Mildred Scheel Cancer Career Center HaTriCS4 University Medical Center Hamburg‐Eppendorf Hamburg Germany; ^3^ Center for Hybrid Nanostructures (CHyN) Universität Hamburg Hamburg Germany; ^4^ Pharmaceutical Institute University of Bonn Bonn Germany; ^5^ Department of Neurosurgery University Medical Center Hamburg‐Eppendorf Hamburg Germany; ^6^ Institute for Advanced Simulation (IAS‐2) Forschungszentrum Jülich Jülich Germany; ^7^ Fleur Hiege Center for Skin Cancer Research University Medical Center Hamburg‐Eppendorf Hamburg Germany; ^8^ Neurology Department Experimental Research in Stroke and Inflammation (ERSI) University Medical Center Hamburg‐Eppendorf Hamburg Germany; ^9^ Experimental Dermatology Medical Faculty Mannheim Heidelberg University Mannheim Germany

**Keywords:** blood flow, circulating tumour cell, heparan sulfate, melanoma, plasma protein, thrombosis, tissue factor

## Abstract

Plasma proteins are increasingly recognized as key regulators of extracellular vesicle (EV) behaviour in circulation. However, von Willebrand factor (vWF)—the largest multimeric glycoprotein in blood and the only blood protein activated by shear—has received little attention in this context. Under elevated shear stress, vWF transitions from a compact globular form to an extended adhesive conformation. Although elevated plasma vWF levels are associated with hypercoagulation in conditions such as malaria, COVID‐19, and cancer, its interactions with EVs or cells under physiological flow have not been systematically investigated. Here, we show that shear‐activated vWF functions as a size‐selective molecular filter that preferentially captures objects smaller than ∼4 µm, including platelets and tumour‐derived EVs, while excluding larger cells. Although intact tumour cells do not directly bind vWF under physiological flow, the coordinated binding of EVs and platelets to extended vWF promotes platelet aggregation, which subsequently traps circulating tumour cells and fosters metastatic dissemination. Our findings reveal a previously unrecognized role of vWF as a shear‐dependent EV‐binding protein that brings EVs and platelets together to enhance coagulation and metastasis. These EV–vWF–platelet aggregates may represent promising biomarkers or therapeutic targets for the prevention of hypercoagulation in cancer and other thrombo‐inflammatory diseases.

## Graphical abstract

Under shear flow, stretched vWF captures platelets and EVs, and TF‐positive EVs amplify platelet activation and aggregation. Although tumour cells do not bind vWF directly, the resulting microthrombi trap circulating tumour cells and promote metastatic progression.

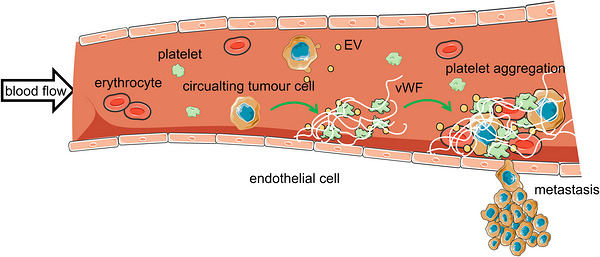

**Abbreviations**
ADAMTS13: a disintegrin and metalloproteinase with a thrombospondin type 1 motif, member 13
AFM: atomic force microscope
EVs: extracellular vesicles
EXT1: exostosin 1
Fad: adhesion force
Fdrag: drag force
GPIbα: glycoprotein Ib alpha
HS: heparan sulfate/heparin
HUVECs: human umbilical vein endothelial cells
NTA: Nanoparticle tracking analysis
PE: Polystyrene
*S*_crit_
: Critical separation
SMFS: Single molecule force spectroscopy
STED: Stimulated emission depletion
TEM: Transmission electron microscopy
TF: Tissue factor
vWF: von Willebrand factor
ΔA1: deletion of the A1 domain
ΔA2: deletion of the A2 domain

## 1 Introduction

The bloodstream hosts various components, among which von Willebrand Factor (vWF) plays a dominant role in coagulation and homeostasis. Synthesized in megakaryocytes and endothelial cells (Wang et al. 2022), vWF represents the largest adhesive ligand in circulation. After synthesis, pro‐vWF undergoes dimerization and multimerization, yielding large multimers that are either constitutively released or stored in the Weibel‐Palade bodies of endothelial cells (EC) or the alpha‐granules of platelets (Wagner et al. 1991). Upon EC activation, typically triggered by vascular stress, injury or inflammation (Huck et al. 2014), vWF multimers are instantaneously released into the bloodstream and unroll into vWF fibres with a length of up to several hundred micrometres (Fu et al. 2017). The ability of vWF to elongate under physiological blood flow conditions is directly linked to the size of the multimer, which defines the target area of the shear force (Schneider et al. 1997). Previous numerical simulations suggest that the size of the vWF multimers is perfectly adjusted to the physiological shear forces acting in our vascular systems to achieve its essential function in primary hemostasis (Rack et al. 2017). This elongation exposes the A1 domain, enabling platelet binding at endothelial activation sites (Feinauer et al. 2021). Previous studies have suggested that the A1 domain vWF interacts not only with platelets but also with erythrocytes, (O'Donnell et al. 2022) leukocytes (Petri et al. 2010) and tumour cells (Bauer et al. 2015; Dhami et al. 2022; Wang et al. 2022). However, the balance between shear stress–induced vWF elongation that promotes adhesion and the shear stress–dependent drag forces that promote cell detachment has not yet been considered (Schneider et al. 2020).

The drag force increases with the size of the bound cell, suggesting that the binding of larger objects such as tumour cells (diameter: > 10 µm) is less likely than the binding of platelets (diameter: 2–4 µm). In contrast, smaller objects such as extracellular vesicles (EVs), may have an increased ability to interact with vWF. EVs are nano‐sized particles released by various cell types, including platelets, leukocytes, and tumour cells. They are delimited by a lipid bilayer (Alberro et al. 2021; Welsh et al. 2024) and carry diverse cargos, such as nucleic acids, proteins, lipids, and small metabolites (Brenna et al. 2020; Welsh et al. 2024). On their surface, EVs possess among others, the prothrombotic tissue factor (TF) (Geddings and Mackman 2013). TF, the key initiator of the extrinsic coagulation cascade, acts in concert with coagulation factors VII and X, leading to thrombin generation and platelet activation (Wang et al. 2023). Recent studies have further demonstrated that cancer‐associated EVs drive thrombosis and metastasis via integrin β2‐mediated platelet activation (Lucotti et al. 2025). In line with that, clinical studies suggested that EVs together with vWF are surrogate markers for hypercoagulation in diseases such as COVID‐19, bacteremia and cancer (Hisada et al. 2022; Sabbagh et al. 2021; Wu et al. 2019). While the individual roles of EVs and vWF in thrombosis have been studied, the combined effects of EV–vWF interactions, shear stress, EV size, and the vWF A1 domain have not yet been investigated.

In this study, we systematically examined how shear stress governs vWF elongation and the capture of circulating components ranging from tumour cells and tumour cell–derived EVs to cell‐like and EV‐sized particles. Microfluidic experiments under physiological flow revealed that vWF is size‐selective, preferentially retaining small particles such as platelets and EVs. TF‐expressing EVs positioned in close proximity to platelets along stretched vWF fibres induce platelet activation and aggregation. This creates a pro‐thrombotic scaffold that promotes the arrest of circulating tumour cells and supports metastatic dissemination.

## 2 Materials and Methods

Detailed methods are provided in the Supporting Information. Original data and analytical methods are available upon reasonable request for reproduction; please contact the corresponding author. All animal experiments were approved by the governmental animal care authorities (‘Behörde für Justiz und Verbraucherschutz, Hamburg’sle project N033/2020). Mice were randomized to experimental groups; data acquisition and analysis were performed blinded to group allocation.

### 2.1 Statistics

Statistical analyses were performed with Python (version 3.9.7) and GraphPad Prism 10. For numerical variables, Student's *t*‐tests were used to compare two groups, and one‐way ANOVA was used for multiple groups, followed by Tukey's post hoc test. Results are presented as mean ± SD, as indicated in the figure legends, *p* ≤ 0.05 was considered statistically significant.

## 3 Results

### 3.1 Binding of Platelets to vWF Depends on Shear Stress

**FIGURE 1:**
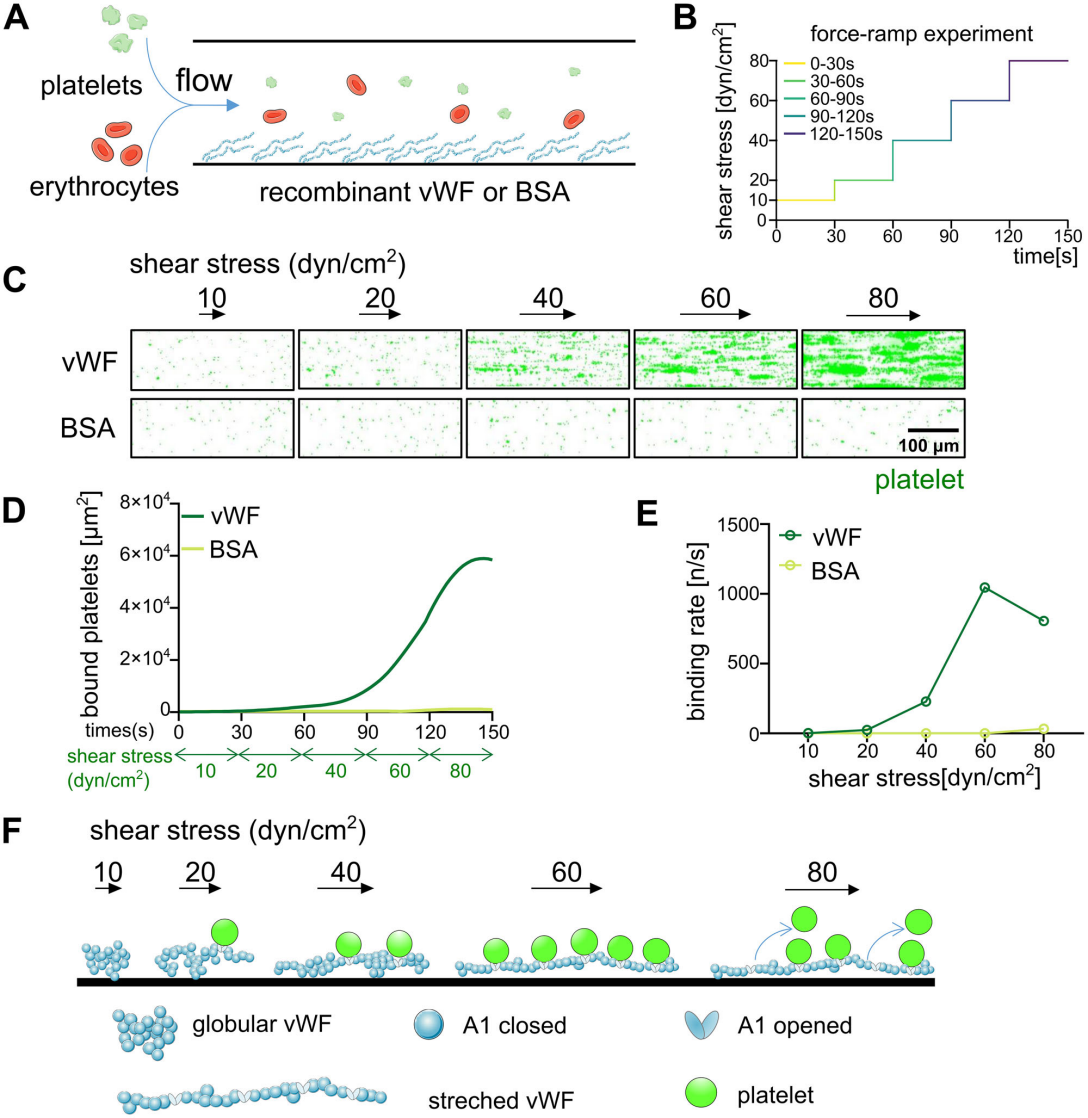
**Binding of platelets to stretched vWF under shear stress**. (**A**) Schematic of the microfluidic experiment. Platelets and erythrocytes (45% haematocrit) were perfused over microfluidic channels coated with recombinant vWF or BSA. (**B**) Force‐ramp experiments were performed with a time‐dependent change of the shear stress. (**C**) Time‐lapse dual‐color fluorescence images of the dynamic interaction of platelets (green) with vWF or BSA over the indicated shear stress range. (**D**) Binding of platelets (green) to vWF over time and increasing shear stress. The x‐axis indicates the time and the distinct force steps (arrows). (**E**) Binding rates of platelets as a function of the applied shear stress, based on data derived from (D). (**F**) Schematic illustrating the stretching of globular vWF under increasing shear stresses, the shear stress‐dependent opening of the A1 domain and the binding of platelets to the open A1 domain. Schematic elements used from Servier Medical Art: https://smart.servier.com/.

To investigate the impact of shear stress on the adhesive properties of vWF under flow, we applied microfluidic devices and live fluorescence microscopy. Washed platelets (300,000/µL) and erythrocytes (45% hematocrit) were perfused over surfaces coated with recombinant vWF or, for control, BSA (Figure 1A). The presence of erythrocytes promoted platelet margination towards the channel wall (Rack et al. 2017). We performed force‐ramp experiments, where shear stress was increased from 10 to 80 dyn/cm^2^ over 150 s (Figure 1B) by a stepwise (30 s steps) increase of the flow rate (Figure S1).

Platelet adhesion to vWF increased with shear stress, whereas adhesion to BSA remained low (Figure 1C; Video S1). On vWF, platelets arranged in a ‘pearl‐on‐string’ pattern, consistent with previous reports and indicative of the elongation of the globular vWF to its stretched conformation (Schneider et al. 2007). Adhesion rose throughout the shear ramp, peaked at ∼140 s (80 dyn/cm^2^), and slightly declined thereafter (Figure 1D), indicating that at high shear the drag force begins to exceed adhesive strength. To better extract the impact of shear stress independent of the time interval, we calculated the binding rate (slope of the adhesion curve within each 30 s segment). Binding rates increased from 20 dyn/cm^2^, peaked at 60 dyn/cm^2^, and decreased at 80 dyn/cm^2^ (Figure 1E). As schematically summarized in Figure 1F, vWF changed from a globular conformation at 10 dyn/cm^2^ to an extensively stretched conformation at 60 dyn/cm^2^ offering binding sites (open A1 domain) to platelets. At 80 dyn/cm^2^, bound platelets or large platelet‐vWF conglomerates (not shown in the schematic drawing) detach as the drag force pulling on the platelets exceeds the adhesion force.

### 3.2 Stretched vWF Favours the Binding of Small Particles

**FIGURE 2:**
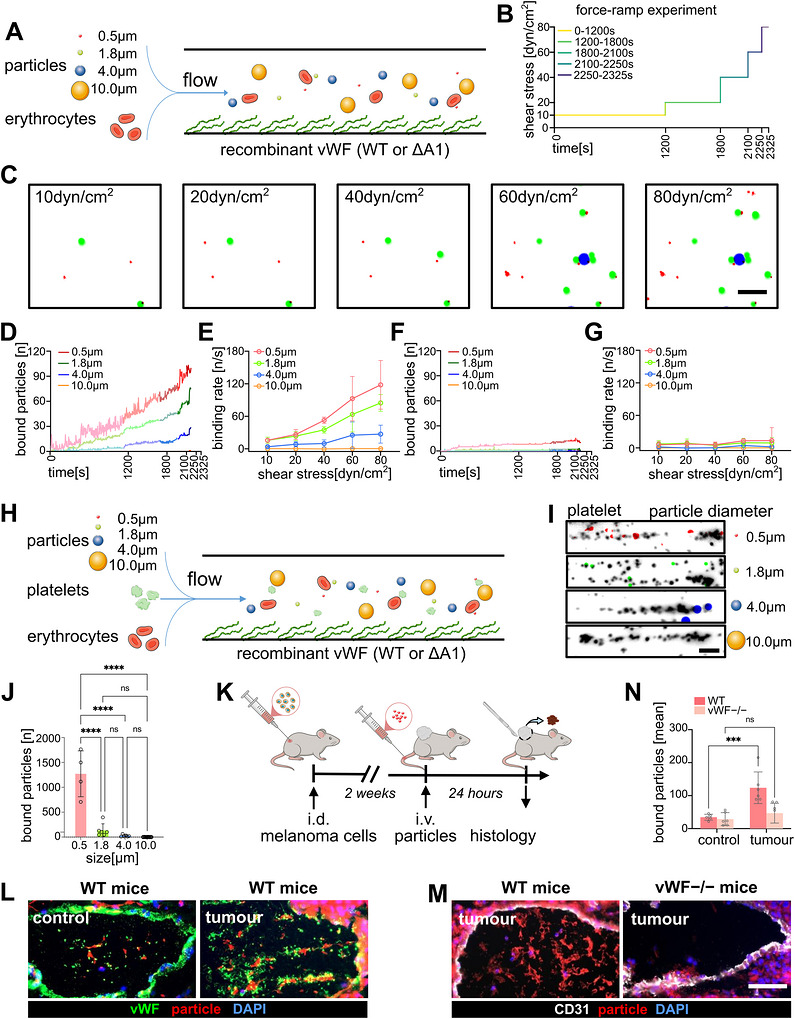
**Binding of particles to stretched vWF under flow conditions**. (**A**) Schematic of the microfluidic experiment: particles of different sizes were perfused through vWF‐coated channels in the presence of erythrocytes. (**B**) Force‐ramp experiments were performed with a time‐dependent change of the shear stress: 10 dyn/cm^2^ (0‐1200 s), 20 dyn/cm^2^ (1201‐1800 s), 30 dyn/cm^2^ (1801‐2100 s), 40 dyn/cm^2^ (2101‐2250 s), 60 dyn/cm^2^ (2251‐2325 s), 80 dyn/cm^2^ (2326‐2364 s). (**C**) Representative images of PE particles bound to WT vWF at the indicated shear stress. Scale bar = 10 µm. (**D**) Particle binding to WT vWF coated surfaces over time and increasing shear stress. Color gradients indicate shear levels (lighter = lower stress, darker = higher stress). (**E**) Binding rates of particles to WT vWF as a function of shear stress. (**F**) Particle binding to ΔA1 vWF coated surfaces over time and increasing shear stress, shown with the same color gradient as in D. (**G**) Binding rates of particles to ΔA1 vWF as a function of shear stress. (**H**) Schematic of the microfluidic experiment: particles of defined sizes were perfused separately through vWF‐coated channels in the presence of platelets and erythrocytes under constant shear stress (60 dyn/cm^2^). (**I**) Particles with diameters of 0.5, 1.8 and 4 µm bound to the channel surface in close proximity to platelets (grey). Particles with a diameter of 10 µm did not bind. Scale bar = 10 µm. (**J**) Quantitative evaluation of fluorescence particle binding in the presence of platelets (*n* = 4). (**K**) Schematic of the mouse experiment. Melanoma cells were intradermally (i.d.) injected into wild‐type (WT) and vWF knockout (vWF−/−) mice. After two weeks, DiD‐labelled nanoparticles (diameter: 157 ± 53 nm) were intravenously (i.v.) administered. 24 h later, the mice were sacrificed, and tumours were analyzed by immunofluorescence staining of the tissue. (**L**) In WT mice, nanoparticles (red) accumulated in the vasculature of tumours in co‐localization with vWF (green) (right). Lack of vWF in non‐tumour control vessel prevented nanoparticle accumulation (left). (**M**) In vWF−/− mice, nanoparticle accumulation was abolished (right). The endothelial cell marker CD31 (white) was used to stain the vascular wall. Nuclei are stained by DAPI (blue). Scale bar = 100 µm. (**N**) Quantification of bound particles in the blood vessels of WT and vWF−/− mice, and control and tumour tissues. (*n* = 6). *** *p* ≤ 0.001, **** *p* ≤ 0.0001, one‐way ANOVA with a Tukey post hoc test. Schematic elements used from Servier Medical Art: https://smart.servier.com/.

To assess how particle size affects interactions with vWF under defined shear stress, we perfused polystyrene (PE) particles (0.5, 1.8, 4, and 10 µm) over vWF‐coated surfaces (Figure 2A). In force‐ramp experiments (Figure 2B), we compared channels coated with either wild‐type vWF or A1 domain–deficient vWF (ΔA1). We have recently shown that cell surface‐exposed heparan sulfate (HS), is a common ligand of the A1 domain (Kalagara et al. 2018; Wang et al. 2022). Surface acoustic wave (SAW) biosensor experiments confirmed binding of HS to WT vWF, whereas binding to ΔA1 vWF was markedly reduced (Figure S2A). To mimic physiological HS‐mediated interactions, the particles used in the following experiments were coated with heparin, a highly sulphated analogue of HS (Figure S2B). Increased negative zeta potentials suggested successful heparin coating of all particles. Overall, all the zeta potentials values were comparable with the exception of the 10 µm particles, which were less negatively charged (Figure S2C). Consistent with the zeta potential data, flow cytometric analysis showed comparable vWF binding for the smaller PE particles, whereas the 10 µm particles displayed reduced binding capacity (Figure S2D,E). In addition, force‐ramp experiments directly demonstrated the significantly increased binding of heparin‐coated PE particles to WT vWF (Figure S2F,G).

As shown in Figure 2C,D, the number of small particles (0.5 and 1.8 µm) bound to WT vWF increased with shear stress. Intermediate‐sized particles (4 µm) showed weak binding above ∼40 dyn/cm^2^, whereas the largest particles (10 µm) did not bind at any of the tested flow conditions. In contrast to the 4 µm particles, binding rates of the 0.5 and 1.8 µm particles increased until the final shear stress of 80 dyn/cm^2^ was reached (Figure 2E). Lack of the A1 domain abolished the binding of the particles (Figure 2F) and no shear stress related change of the binding affinity was measured (Figure 2G).

Next, we performed experiments in the presence of both platelets and PE particles (Figure 2H). Particles bound in close proximity to adherent platelets (Figure 2I), with highest binding observed for 0.5 µm particles and no detectable adhesion for 10 µm particles (Figure 2J).

To test whether particles bind to vWF fibres in vivo, we injected non‐toxic fluorescent nanoparticles intravenously into tumour‐bearing mice (Figure 2K). The nanoparticles (∼0.2 µm in diameter (von Palubitzki et al. 2020)) fell within the size range retained by vWF. In tumour vasculature, endothelial cells release vWF into the vessel lumen while levels of the vWF‐cleaving ADAMTS13 (a disintegrin and metalloproteinase with a thrombospondin type 1 motif, member 13) are reduced, resulting in persistent vWF fibre formation (Bauer et al. 2015). Fluorescent nanoparticles accumulated along vWF fibres in tumour vessels of wild‐type mice (Figure 2L, right), but not in healthy control vessels lacking vWF fibres (Figure 2L, left). No accumulation was observed in vWF‐deficient mice (Figure 2M, right). Quantitative image analysis (Figure 2N) confirmed a significant association between vWF fibre formation and nanoparticle binding.

### 3.3 Theoretical Calculations of Particle Binding to vWF‐Coated Surfaces

**FIGURE 3:**
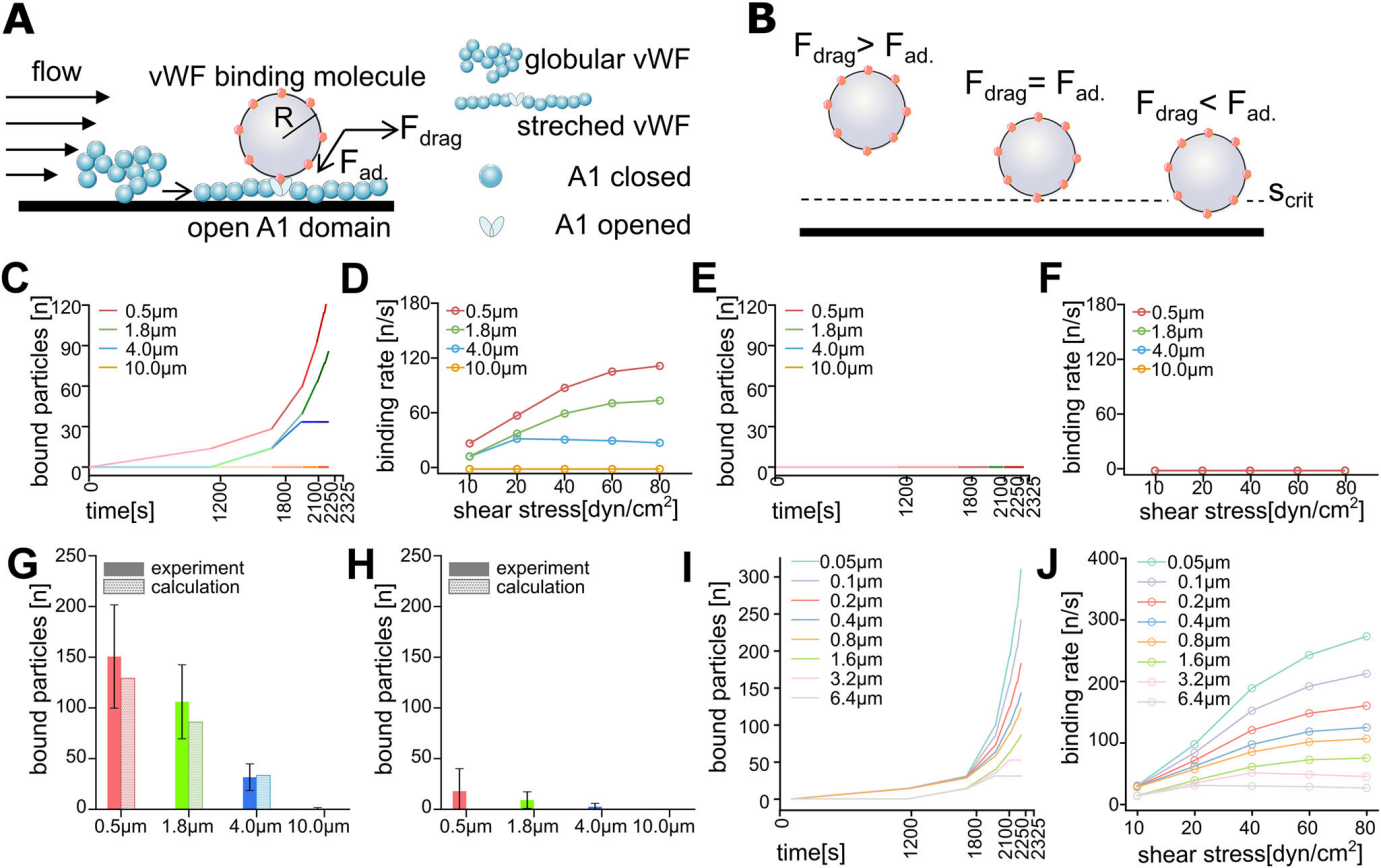
**Theoretical calculation of particle binding to vWF under shear stress**. (**A**) Schematic illustration of particle binding to vWF fibres under flow. Particle binding depends on the size of the particle (particle radius, R), the drag force (*F_drag_*) pulling at the particle and the adhesion force (*F_ad_*). (**B**) The balance between *F_drag_* and *F_ad_* promote particle adhesion. At a separation above *s_crit_*, *F_drag_* exceeds *F_ad_* and particles detach. At a separation below *s_crit_*, *F_drag_* is smaller than *F_ad_* and particles bind. (**C**) Calculated binding of particles to WT vWF‐coated surface over time and increasing shear stress. (**D**) The particle binding rate derived from the data shown in (C) as a function of shear stress. (**E**) Calculated particle binding to ΔA1 vWF‐coated surface over time and increasing shear stress. (**F**) Corresponding binding rates from (E) as a function of shear stress. (**G**, **H**) Comparison of calculated and experimental data showing total bound particles on WT (G) and ΔA1 (H) vWF‐coated surfaces at the final time point. (**I**) Calculation of particle binding to WT vWF over a broader particle size range. (**J**) Calculated binding rates of particles to WT vWF; related to the data shown in (I).

To complement our experimental findings, we performed theoretical calculations of particle binding to vWF under shear flow. The model, described in detail in the Supporting Information and Figures S3–S4, considers the balance between the adhesive force of a particle near the vWF‐coated surface (*F_ad_*) and the drag force exerted by the flow (*F_drag_*) (Figure 3A). We assumed that at a critical separation distance (*s_crit_*), *F_drag_* equals *F_ad_*. Particles located closer than *s_crit_* adhere to the surface, whereas particles above this threshold cannot bind (Figure 3B).

Since *F_drag_* depends on both the applied shear stress and particle diameter, we calculated binding across different shear conditions and sizes. Binding increased over time (Figure 3C), and, consistent with our experimental data (Figure 2), small particles (0.5 and 1.8 µm) adhered most efficiently, whereas 10 µm particles did not bind. At shear stresses below 40 dyn/cm^2^, binding of 0.5 and 1.8 µm particles increased with shear, indicating that *F_ad_* exceeds *F_drag_* (Figure 3D). At higher shear stresses (>60 dyn/cm^2^), a plateau was reached, suggesting force equilibrium. Although 4 µm particles occasionally adhered (Figure 3C), their binding was largely insensitive to shear (Figure 3D).

We repeated the calculations for ΔA1 vWF‐coated surfaces (Figure 3E,F). In agreement with experimental observations (Figure 2F,G), no particles bound under any condition. To facilitate comparison between theory and experiments, we quantified bound particles at the final time point. Figure 3G summarizes results for WT vWF surfaces, and Figure 3H shows those for ΔA1 vWF.

Overall, the calculations reproduced the main experimental trends and support the idea that stretched vWF preferentially captures smaller particles, whereas larger particles fail to bind stably.

Our experiments showed that 4 µm particles bind only weakly to vWF, indicating that this size is near the threshold for adhesion. To refine this threshold, we extended our theoretical calculations to additional particle sizes (Figure 3I). Particles of 1.6 µm still showed increasing adhesion at high shear stress, whereas adhesion of 3.2 and 6.4 µm particles declined above ∼40 and ∼20 dyn/cm^2^, respectively (Figure 3J). These data indicate that the critical size for stable adhesion lies between 1.6 and 3.2 µm. Notably, platelets, fall within this range, suggesting that their size represents an optimal balance between shear‐dependent adhesion and cargo capacity in the present system.

### 3.4 Characterization of Melanoma Cell‐Derived EVs

Based on our results indicating that smaller particles bind more efficiently to stretched vWF under shear, we hypothesized that EVs, rather than intact tumour cells, are capable of binding to vWF. To test this, we isolated and characterized EVs from B16F10 metastatic melanoma cells according to MISEV guidelines (Welsh et al. 2024), which are known to release large quantities of EVs enriched in the HS‐exposing proteoglycan glypican (García‐Silva et al. 2021).

**FIGURE 4:**
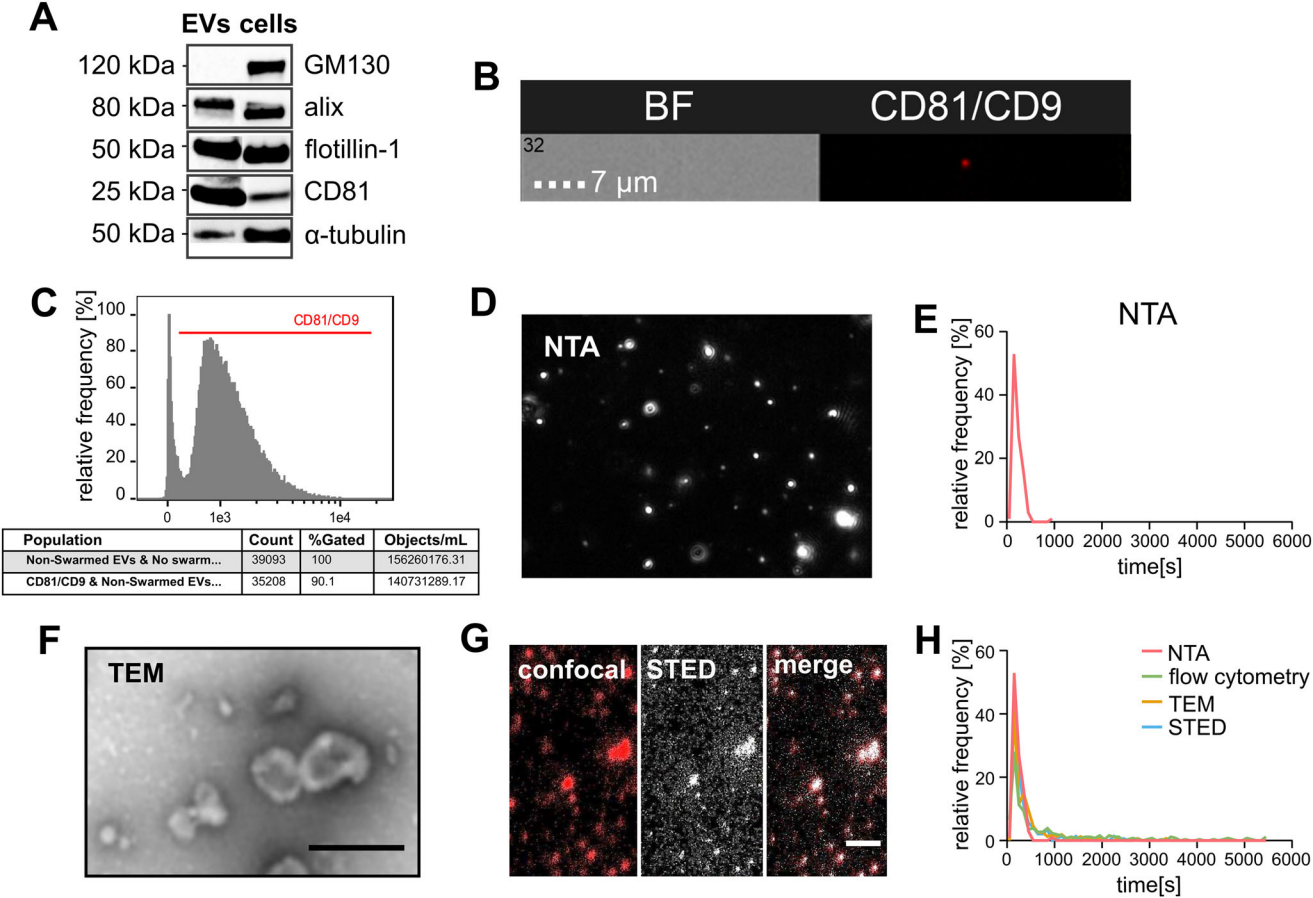
**Characterization of B16F10 melanoma cell‐derived EVs**. (**A**) Western blot analysis of EVs and B16F10 cells. CD81 (22 kDa), flotillin‐1 (48 kDa) and alix (96 kDa) were detected in EVs and cells. The Golgi resident protein GM130 served as a non‐EV marker and was only detected in cells, α‐Tubulin served as a loading control. (**B**) Representative IFCM image of EVs stained for the tetraspanins CD81/CD9. Brightfield (BF) and CD81/CD9 fluorescence are shown. Scale bars = 7 µm. (**C**) Histogram of IFCM showing that ∼90% of non‐swarmed EVs were positive for CD81/CD9. (**D**) Representative NTA image and determined size distribution of EVs. (**E**) The particle diameter ranged from 118.8 nm to 324.9 nm, based on the 10th to 90th percentile NTA measurements. The mean diameter of the EVs was 213.9 ± 4.5 nm. (**F**) Representative transmission electron microscopy (TEM) image. Scale bar = 250 nm. (**G**) Confocal laser scanning microscopy (CLSM) image (red), STED image (white) and merge of both channels to indicate super‐resolution of the STED. EVs were labelled with the lipophilic tracer DiD. Scale bar = 2 µm. (**H**) Size distributions of EVs obtained by NTA, flow cytometry, TEM and STED microscopy.

EV markers were characterized by Western blot, with whole‐cell lysates as reference (Figure 4A). Alix (96 kDa) and the membrane‐bound flotillin‐1 (48 kDa) were present in the isolated EVs and the cell lysate. The Golgi resident protein GM130 (a marker for non‐EV contamination) was detected only in cell lysates. In contrast, the tetraspanin CD81 was enriched in EVs, confirming high purity of the preparations. In addition, the tetraspanins CD81 and CD9 were also present when analysed by IFCM, representing 90% of the melanoma EVs (Figure 4B,C). Size and concentration of EVs were determined by nanoparticle tracking analysis (NTA) (Figure 4D). The EV concentration was 1.61 × 10^9^ ± 3.74 × 10^7^ particles/mL with a mean diameter of 213.9 ± 4.5 nm and a dominant population at ∼165 nm (Figure 4E). The detection range of NTA is limited to the size range between 50 and 1000 nm (Dragovic et al. 2011); therefore, we additionally measured the size of the EVs by flow cytometry, transmission electron microscopy (TEM) (Figure 4F) and super‐resolution stimulated emission depletion (STED) microscopy (Figure 4G). All techniques yielded consistent size distributions, with the majority of EVs between 118.8 and 324.9 nm (Figure 4H). As this lies within the vWF capture range, EVs are likely to bind efficiently under shear. Melanoma cells (>10 µm), by contrast, exceed this range and are expected to show negligible adhesion.

### 3.5 EVs But Not Whole Cells Bind to Stretched vWF

**FIGURE 5:**
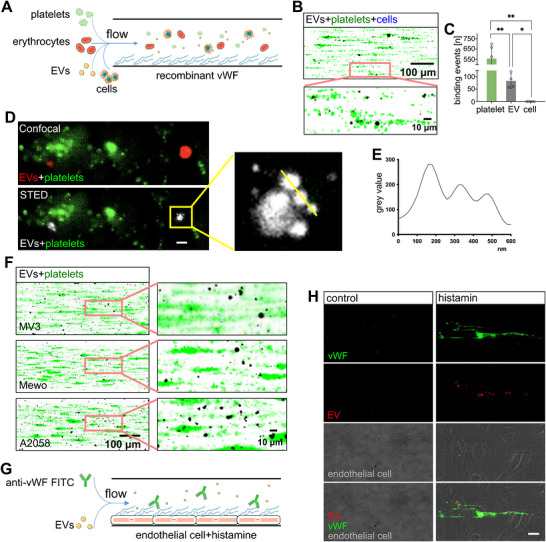
**Binding of melanoma cell derived‐EVs to stretched vWF under flow**. (**A**) Schematic of the microfluidic experiment: melanoma cells and melanoma cell‐derived EVs were perfused through vWF‐coated channels in the presence of platelets and erythrocytes. (**B**) EVs (black) and platelets (green) bind to the vWF‐coated surface. Pearl‐on‐string‐like arrangement indicate the interaction of platelets and EVs with vWF fibres. Bound B16F10 cells (principally labelled in blue) were not detected. (**C**) Quantification of bound platelets, EVs and cells (*n* = 5). * *p* ≤ 0.05, ** *p* ≤ 0.01 one‐way ANOVA with Tukey post hoc test. (**D**) Confocal laser scanning microscopy (CLSM) image (upper), and STED image (down) showed super‐resolution of EV aggregates (white) and platelets (green) bound to vWF. Scale bar = 1 µm. (**E**) Size distributions of EVs in EV aggregates derived from the cross‐section indicated on STED image (yellow line). (**F**) EVs (black) isolated from different melanoma cell lines (MV3, Mewo, A2058) bound to the vWF‐coated surfaces. (**G**) Schematic of the microfluidic experiment: HUVECs were coated on the bottom of channels. To release vWF, HUVECs were stimulated with histamine. EVs and FITC‐conjugated antibodies directed against vWF were perfused through the flow channels. (**H**) After histamine stimulation and the release of vWF, EVs (red) bound directly to vWF fibres (green). No EVs bound directly to the HUVECs (bright field). Scale bar = 20 µm. Schematic elements used from Servier Medical Art: https://smart.servier.com/.

To test whether EVs but not whole cells bind to vWF fibres, we perfused vWF coated surfaces with B16F10 cell‐derived EVs, B16F10 cells, platelets and erythrocytes (45% haematocrit) (Figure 5A). As shown in Figure 5B, platelets form a pearl‐on‐string configuration indicating their interaction with vWF fibres. In contrast, B16F10 cells did not attach to the surface in any of our experiments. Instead, we observed a significant accumulation of EVs in close proximity to platelets (Figure 5B). A direct comparison revealed that EVs bound to vWF fibres less efficiently than platelets (Figure 5C), although EVs are smaller than platelets—and thus according to our theoretical model should bind more efficiently. This discrepancy occurs because (i) many individual EVs (∼50‐200 nm) fall below the resolution limit of conventional fluorescence microscopy, and (ii) when EVs cluster in close proximity, they are optically resolved as single fluorescent spots—both leading to systematic underestimation of EV binding events. In line with this, STED microscopy revealed that EVs appearing as single large spots in confocal laser scanning microscopy were in fact conglomerates of multiple EVs (Figure 5D). Line‐profile analysis confirmed that the resolved EV signals were within the expected EV size range of approximately 50–200 nm (Figure 5E). Additionally, and as further outlined in the discussion, platelets are much softer than EVs and deform under shear stress (Calò et al. 2014; Liu et al. 2019; Nesbitt et al. 2009). This deformation, which was not considered in our calculation, increases the area of adhesion and in turn promotes the interaction of platelets with vWF.

EVs are produced by most mammalian cells and we confirmed that EVs from the human melanoma cell lines MV3, MeWo and A2058 also bind to vWF in close proximity to platelets (Figure 5F). To exclude the possibility that EV binding is influenced by platelets, we repeated the experiment without platelets. EV Binding to vWF was still observed and significantly reduced upon addition of ADAMTS13 (Figure S5).

To demonstrate direct and specific EV binding to vWF under physiological, low shear stress conditions (5 dyn/cm^2^), we perfused B16F10‐derived EVs over human umbilical vein endothelial cell (HUVEC) monolayers (Figure 5G). Release of vWF from endothelial cells was induced by histamine stimulation. During perfusion, vWF fibres were labelled in real time by adding a fluorophore‐conjugated anti‐vWF antibody to the medium. EVs bound directly to the released vWF fibres, whereas no EV adhesion was detected on non‐stimulated HUVECs (Figure 5H).

### 3.6 EVs Binding to vWF Depends on the A1 Domain and HS

**FIGURE 6:**
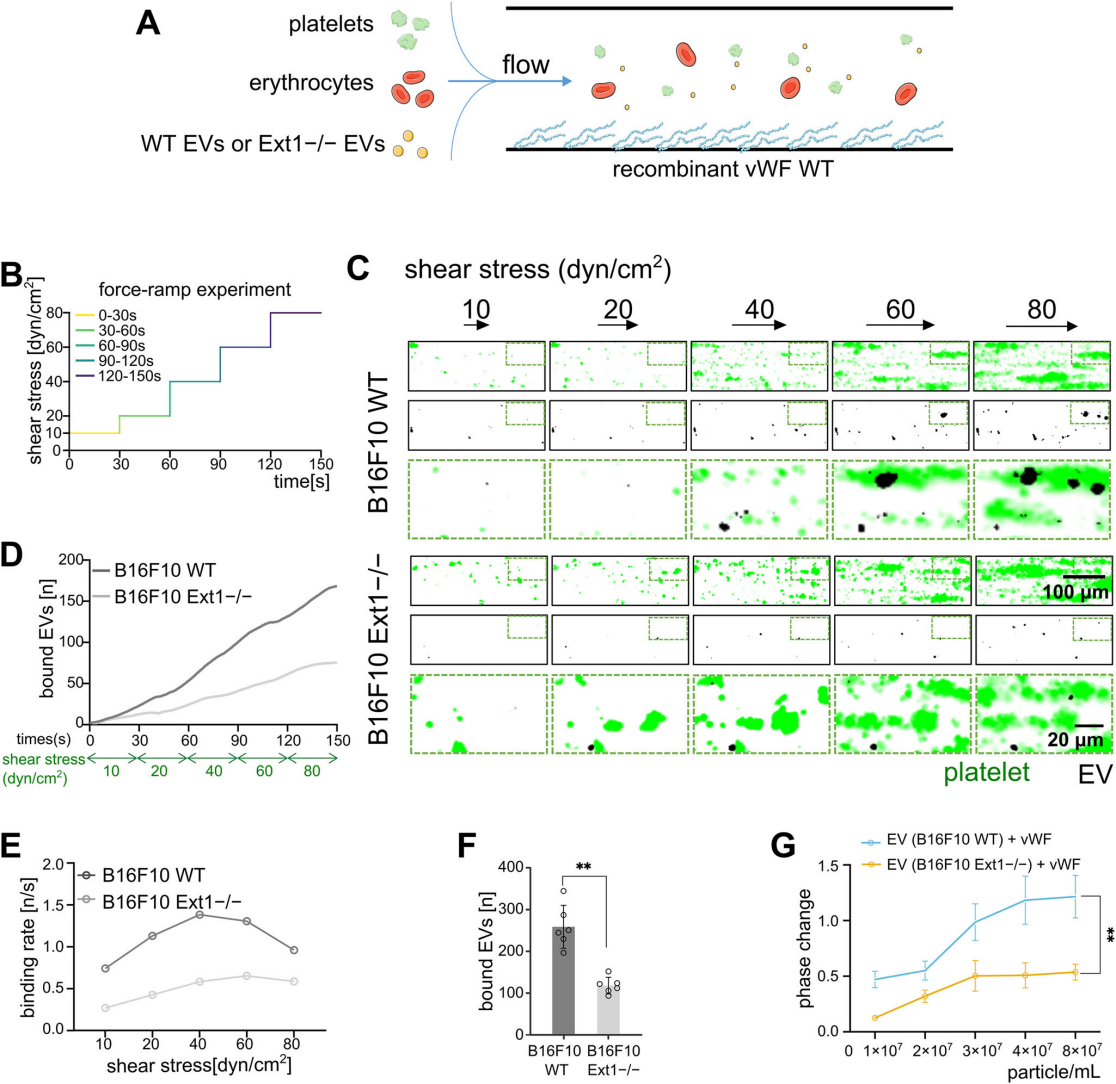
**Binding of melanoma cell derived EVs to stretched vWF depends on HS**. (**A**) Schematic of the microfluidic experiment: EVs derived from WT and Ext1−/− cells were perfused through microfluidic channels coated with recombinant WT vWF in the presence of platelets and erythrocytes. (**B**) Force‐ramp experiments were performed with a time‐dependent change of shear stress. (**C**) Time‐lapse dual‐color fluorescence images showing platelets (green) and EVs (black) interaction with vWF at indicated shear stresses. Merged images highlight regions of interest (dashed boxes). (**D**) Binding of WT or Ext1−/− EVs (black) to vWF over time and increasing shear stress. (**E**) Binding rates of EVs to vWF‐coated surfaces indicated that EV binding increases with increasing shear stress until 40 dyn/cm^2^ was reached. (**F**) Quantification of bound EVs at the endpoint of the experiment (*n* = 7). **p ≤ 0.01 Student's *t* test. (**G**) SAW biosensor measurements showed that WT EVs bound stronger to immobilized vWF than Ext1−/− EVs, as indicated by a larger phase shift (*n* = 3). Schematic elements used from Servier Medical Art: https://smart.servier.com/.

HS at the surface of cells is a relevant ligand for the A1 domain of vWF (Wang et al. 2022). To prove whether HS also contributes to EV‐vWF binding, we isolated EVs from B16F10 cells lacking exostosin 1 (EXT1, B16F10 Ext1−/− cells). EXT1 is a key enzyme of the HS biosynthesis and its knockout abolishes cell‐surface HS expression (Wang et al. 2022; Grossdorf et al. 2025). As shown in Figure 6A, we perfused vWF‐coated surfaces either with EVs derived from WT cells or Ext1−/− cells together with platelets and erythrocytes. EV binding was assessed using a force‐ramp experiment (Figure 6B). Compared to EVs from WT cells, HS‐deficient EVs showed markedly reduced binding to vWF (Figure 6C). For both EV types, the number of adherent EVs increased over time and with higher shear stress (Figure 6D). However, HS‐deficient EVs exhibited a substantially lower binding rate (Figure 6E), and by the end of the experiment, their total binding was two‐fold lower than that of WT EVs (Figure 6F). Additionally, blockage of the A1 domain by heparin largely reduced EV and platelets binding (Figure S6A‐C).

To further verify the role of the A1 domain in HS‐mediated EV binding, we tested ΔA1 vWF. Deletion of the A1 domain lowered the binding rate and the total number of bound EVs (Figure S6D–H). Moreover, addition of ADAMTS13 prevented EV accumulation at the channel surface (Figure S6I‐K). Taken together, our experiments shown in the Figures 6, Figure S5 and Figure S6 indicate that, under shear, EV binding to vWF is mediated by HS engagement of the A1 domain.

Platelets were included in all perfusion experiments as an internal control. ADAMTS13 did not affect platelet binding at shear stresses below 60 dyn/cm^2^, but reduced adhesion at higher shear (Figure S6L,M). A similar decrease was observed on ΔA1 vWF–coated surfaces, although binding was not completely abolished, suggesting partial compensation by platelet‐derived vWF. As expected, platelet adhesion was comparable between EVs from wild‐type and HS‐deficient cells (Figure S6L,M).

To further assess the specificity and strength of the EV‐vWF interaction, we used SAW biosensor assay to quantify the real‐time binding kinetics of B16F10 WT and Ext1−/− EVs to WT vWF‐coated surfaces. Upon the addition of WT EVs, a pronounced and dose‐dependent phase shift was observed, indicating strong binding to vWF (Figure 6G). In contrast, Ext1−/− EVs induced a significantly lower phase shift, reflecting a markedly reduced binding affinity. Parallel experiments with ΔA1 vWF‐coated surfaces revealed diminished phase shifts for WT EVs (Figure S6N), demonstrating that A1‐domain deletion substantially reduced their binding.

### 3.7 Binding of EVs to vWF Fibres Supports Platelet Aggregation

**FIGURE 7:**
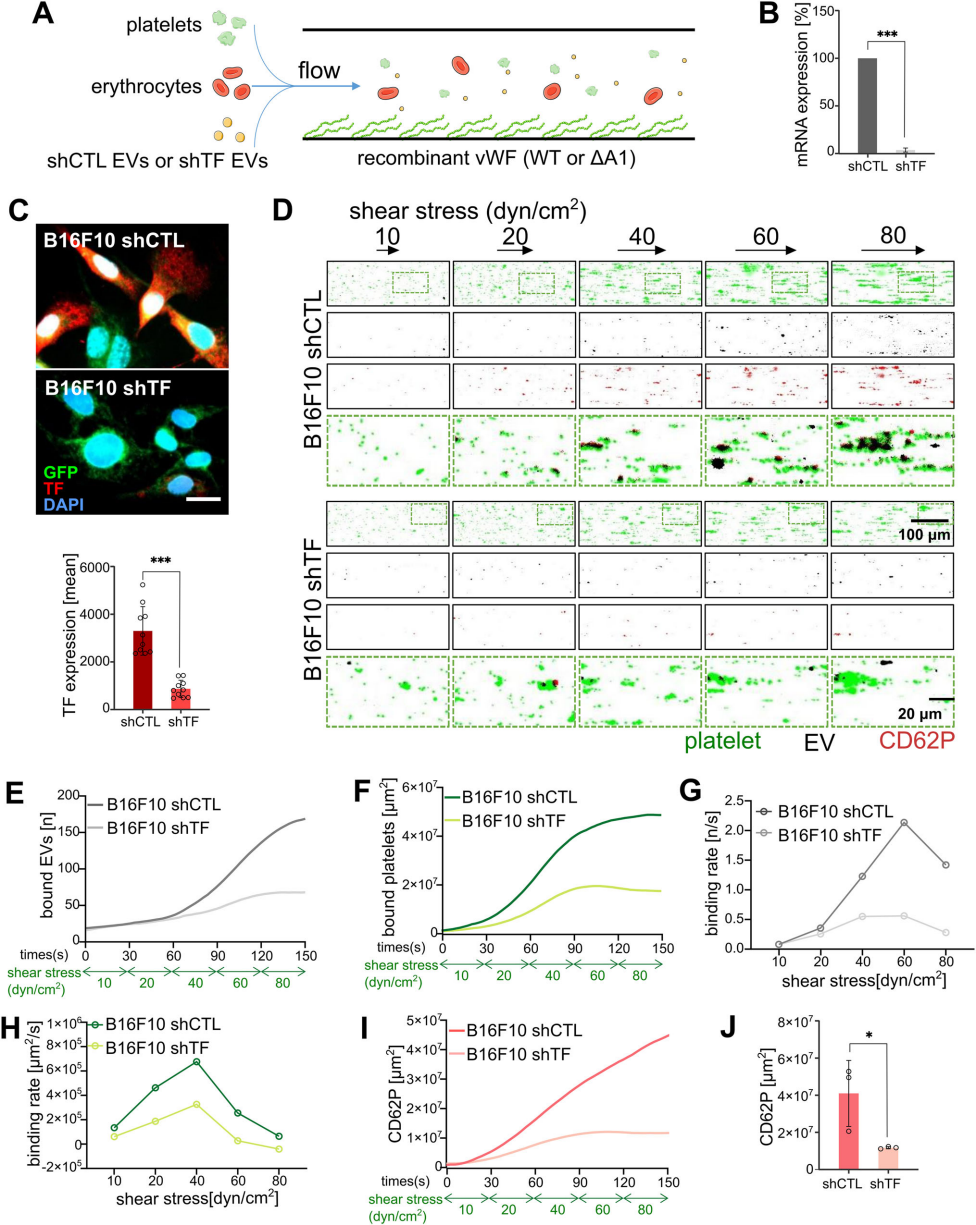
**Binding of EVs to vWF induces platelet activation**. (**A**) Schematic of the microfluidic experiment: EVs isolated from control (shCTL) and TF knockdown cells (shTF) were perfused through vWF‐coated microfluidic channels in the presence of platelets, plasma and erythrocytes. (**B**) TF expression was significantly reduced in B16F10 shTF cells compared to shCTL controls, as measured by qPCR (*n* = 3). (**C**) Immunofluorescence staining confirmed reduced TF expression in shTF cells (n = 10). Scale bar = 20 µm. (**D**) Time‐lapse dual‐color fluorescence images showing platelet (green) and EV (black) binding to vWF at varying shear stresses. CD62P staining (red) was used to measure platelet activation. Merged images include zoomed‐in regions (dashed boxes). (**E**) Binding of shCTL and shTF EVs to vWF‐coated surfaces over time and increasing shear stress. (**F**) Binding of platelets to vWF‐coated surfaces in the presence of shCTL or shTF EVs over time and increasing shear stress. (**G**) Binding rates of shCTL and shTF EVs to vWF, derived from (E), as a function of shear stress. (**H**) Platelet binding rates in the presence of shCTL or shTF EVs, derived from (F), as a function of shear stress. (**I**) Platelet activation over time and increasing shear stress, comparing channels with shCTL versus shTF EVs. (**J**) Quantification of EV‐induced platelet activation at the end of the experiment (*n* = 3). * *p* ≤ 0.05, *** *p* ≤ 0.001, Student's *t* test. Schematic elements used from Servier Medical Art: https://smart.servier.com/.

Due to the simultaneous binding of EVs and platelets along the vWF fibres, TF is positioned in close proximity to the platelets. In the presence of plasma, TF produces thrombin, a strong activator of platelets (Lima et al. 2011). To study the potential impact of TF on platelet activation we added plasma and CaCl_2_ into the perfusing medium (Figure 7A). Plasma from healthy donors contains physiological levels of ADAMTS13 cleaving stretched vWF inside the A2 domain. To better mimic the pathophysiological reduction of ADAMTS13 (Bauer et al. 2015) activity observed in cancer patients, we used vWF lacking the A2 domain (ΔA2 vWF). Compared to WT vWF, ΔA2 vWF was resistant to proteolysis and vWF fibre formation. Consistently, EV and platelet binding was more pronounces (Figure S7A).

Then, we compared the effect of EVs derived from TF knockdown cells (B16F10 shTF) and EVs derived from control cells (B16F10 shCTL). The shRNA‐induced knockdown of TF was quantified by qRT‐PCR and immune fluorescence staining (Figure 7B,C). In addition, TF‐deficient (TF−/−) cells were generated and characterized independently (Figure S7B). TF abundance and the relative fraction of TF‐positive EVs were further analysed by imaging flow cytometry (IFCM). As shown in Figure S7C, TF was undetectable on TF−/− EVs, whereas WT EVs showed a significantly higher TF signal. On average, 1.39 ± 0.59% of WT EVs were TF‐positive (Figure S7D‐G).

Figure 7D shows representative images taken during the microfluidic experiment at different shear stresses. Consistent with our previous experiments, increasing shear stress increased the binding of EVs (Figure 7E) and platelets (Figure 7F) to vWF. However, calculating the corresponding binding rates revealed that the critical shear stress for EV and platelet binding was lower compared to experiments without plasma (Figure 7G,H). In our previous plasma‐free experiments (Figures 1 and 6), the critical shear stress was ∼60 dyn/cm^2^; at which, adhesion plateaued and the binding rate began to decline. Here, in the presence of plasma, the critical shear decreased to ∼40 dyn/cm^2^. This shift agrees well with the formation of larger platelet aggregates. The reduction in critical shear stress was more pronounced in channels perfused with the EVs from B16F10 shCTL cells than in channels containing the EVs from B16F10 shTF cells. Consistently, platelet aggregates increased more rapidly and reached larger sizes in the control cell group (Figure 7D). To confirm platelet activation, we added a fluorescence‐conjugated antibody directed against P‐selectin (CD62p) to the perfusing medium. As shown in Figure 7D and Video S2, platelets displayed a time‐dependent increase in surface P‐selectin. P‐selectin presentation was more prominent in channels containing the EVs from the shCTL cells (Figure 7I,J). In addition to platelet activation, FITC‐conjugated fibrinogen accumulated in the presence of TF‐positive EVs suggesting the thrombin‐mediated formation of fibrin (Figure S7H,I). Platelet activation and the formation of fibrin and larger platelet aggregates indicate blood clotting within the channels resembling the situation of microvessels in patients with hypercoagulation (Goertz et al. 2016; Kobayashi et al. 2019; Lima et al. 2011).

To further assess the role of TF, we compared EVs from B16F10 and A2058 melanoma cells, the latter expressing significantly less TF (Figure S8A–C; EV characterization in Figure S8D). In microfluidic assays, EV binding to vWF and the final platelet‐covered area were similar for both EV types (Figure S8E–G). However, B16F10 EVs promoted earlier platelet adhesion at lower shear (Figure S8H,I) and induced markedly larger platelet aggregates than A2058 EVs (Figure S8J,K).

### 3.8 Activated Platelets Trap Tumour Cells in EV‐Induced Aggregates and Promote Metastasis

**FIGURE 8:**
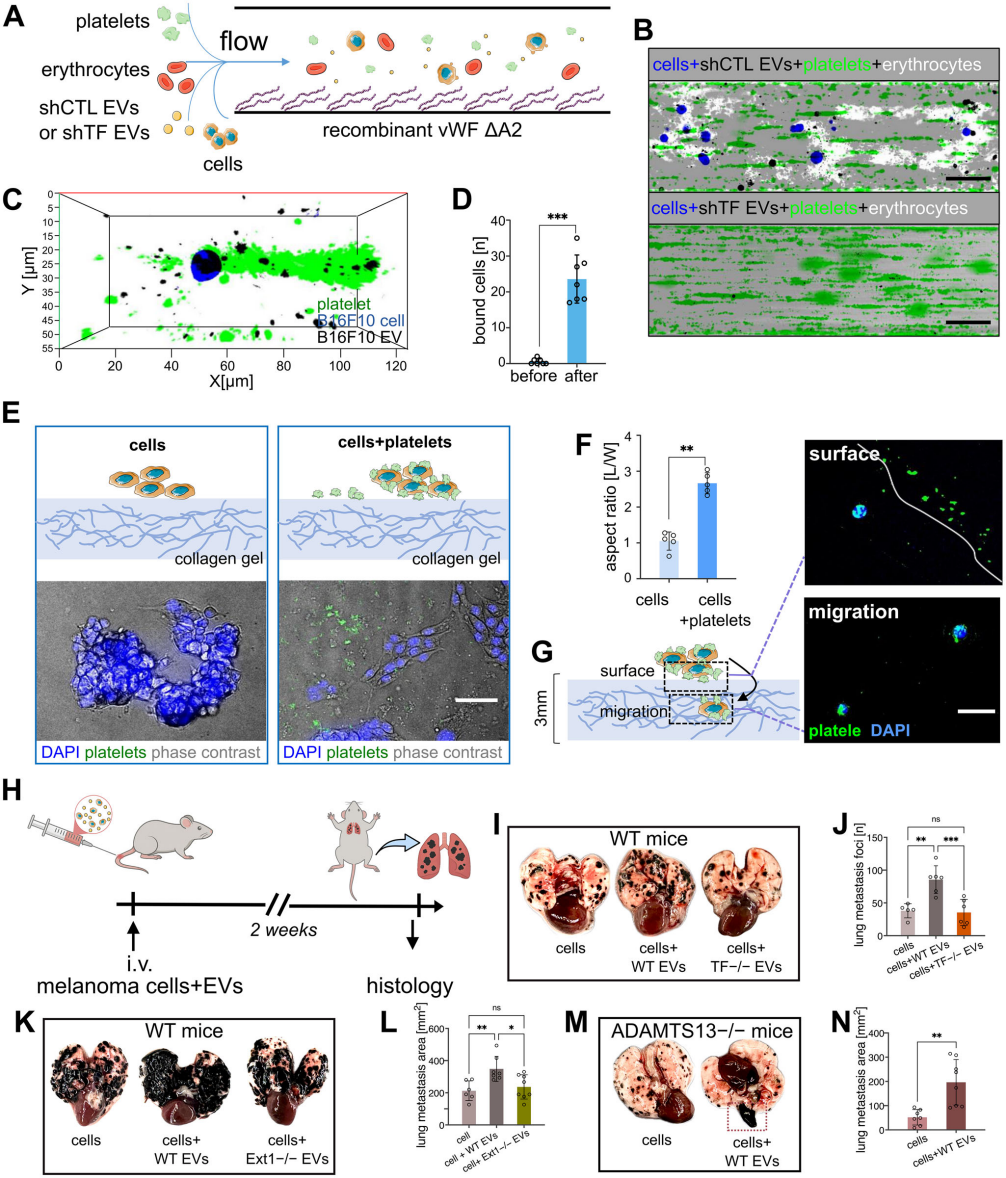
**Conglomerates of EVs, platelets and vWF trap tumour cells**. (**A**) Schematic of the microfluidic experiment: B16F10 melanoma cells and EVs (from shCTL or shTF cells) were perfused through vWF‐coated channels in the presence of platelets, plasma, and erythrocytes. (**B**) B16F10 cells (blue) bound to vWF in channels containing EVs isolated from shCTL B16F10 cells. Binding of the melanoma cells was associated with the aggregation of erythrocytes (white). No B16F10 cells or erythrocytes accumulated in channels perfused with EVs isolated from shTF B16F10 cells. Scale bar = 100 µm. (**C**) 3D rendered image showing the interaction of a melanoma cell (blue) with platelets (green) and EVs (black). The image was generated from optical sections acquired by structured illumination microscopy. (**D**) Quantification of trapped B16F10 cells before and after EV‐platelet conglomerate formation (*n* = 7). *** *p* ≤ 0.001 Student's *t* test. (**E**) B16F10 cells were seeded on collagen gels with or without thrombin activated platelets. In the absence of platelets, melanoma cells were round shaped and formed clusters. In the presence of platelets, melanoma cells showed an elongated shape and no clustering. Scale bar = 50 µm. (**F**) Quantification of the aspect ratio of B16F10 cells in the presence or absence of activated platelets. ** *p* ≤ 0.01 Student's *t* test. (**G**) Migration of B16F10 cells incubated with platelets from the surface to the centre of the collagen gel. Representative fluorescence images show the gel surface (surface of the gel is indicated by the white line) and the central part of the gel (migration). Platelets (green) accumulated at the gel surface and around single melanoma cells (blue). Scale bar = 50 µm. (**H**) Schematic of the mouse experiment. Melanoma cells and EVs were intravenously (i.v.) injected into wild‐type (WT) and ADAMTS13 deficient (ADAMTS13−/−) mice. After two weeks, lung metastasis was quantified. (**I, K, M**) Representative images of metastatic lungs of wild type (WT) mice (I) and ADAMTS13 deficient mice (K). (**J, L, N**) Quantification of metastatic foci (*n* = 5–8) or area. * *p* ≤ 0.05, ** *p* ≤ 0.01, *** *p* ≤ 0.001, one‐way ANOVA with Tukey post hoc test (J, L). ** *p* ≤ 0.01, Student's *t* test (N).

We next tested whether the EV–vWF–platelet coagulation axis can trap circulating tumour cells. Based on the experimental setup shown in Figure 7, we added WT B16F10 melanoma cells into the perfusing medium (Figure 8A). While our data, shown in Figure 5, indicate that melanoma cells do not bind directly to stretched vWF, we now observed their entrapment within large thrombus‐like aggregates composed of platelets, EVs and erythrocytes (Figure 8B,C). EV‐induced platelet aggregation significantly increased the number of trapped tumour cells (Figure 8D).

Previous studies have indicated that platelets support tumour cell dissemination and may aid vascular escape (Haemmerle et al. 2018). To follow the effect of platelet‐mediated entrapment, we seeded B16F10 cell on a soft collagen matrix with or without activated platelets. After an incubation time of 48 h the morphology and migration of the cells were measured by fluorescence microscopy (Figure 8E). In the absence of platelets, B16F10 cells formed clusters and had a round morphology, indicating poor adhesion and reduced viability. In contrast, cells interacting with activated platelets displayed an elongated morphology, indicating adherent and viable cells. In platelet free experiments, cells had an aspect ratio (length/width) of 1.1 ± 0.10, whereas platelet‐associated cells had a significantly increased ratio of 2.7 ± 0.15 (Figure 8F). In addition, only platelet‐associated cells migrated into the collagen gel (Figure 8G). During their migration, tumour cells escaped from the clot but retained a pericellular ‘platelet shell’, which was detectable even in deeper regions of the collagen matrix.

To assess the role of TF and vWF in vivo, we analysed the impact of EVs on metastasis. EVs derived from WT or TF−/− B16F10 cells were co‐injected with WT B16F10 cells into recipient mice, whereas control animals received WT cells alone (Figure 8H). In WT mice, co‐injection of WT EVs and WT cells significantly enhanced metastasis compared to the co‐injection of WT cells and TF−/− EVs or injection of WT cells only (Figure 8I,J). These data are consistent with our *in vitro* assays indicating that EVs activate platelets through TF to promote metastatic seeding. To further probe the role of vWF‐dependent EV binding, we compared WT EVs with Ext1−/− EVs. Consistent with their reduced binding to vWF, Ext1−/− EVs promoted less metastasis than WT EVs (Figure 8K,L). To further evaluate the contribution of vWF fibres, parallel experiments were performed in ADAMTS13‐deficient (ADAMTS13−/−) mice. In ADAMTS13−/− mice, the number of metastatic foci in the lungs was similar in the groups receiving cells with WT EVs and the group receiving only cells (Figure S9A). However, the overall metastatic burden—measured as total metastatic area—was significantly higher in ADAMTS13−/− mice that received WT cells together with EVs (Figure 8M,N). Notably, several exceptionally large metastatic foci were observed in the lungs of the corresponding animals (marked with red dashed boxes in Figure 8M) suggesting the entrapment of multiple tumour cells in thrombi prior to the extravasation.

**FIGURE 9:**
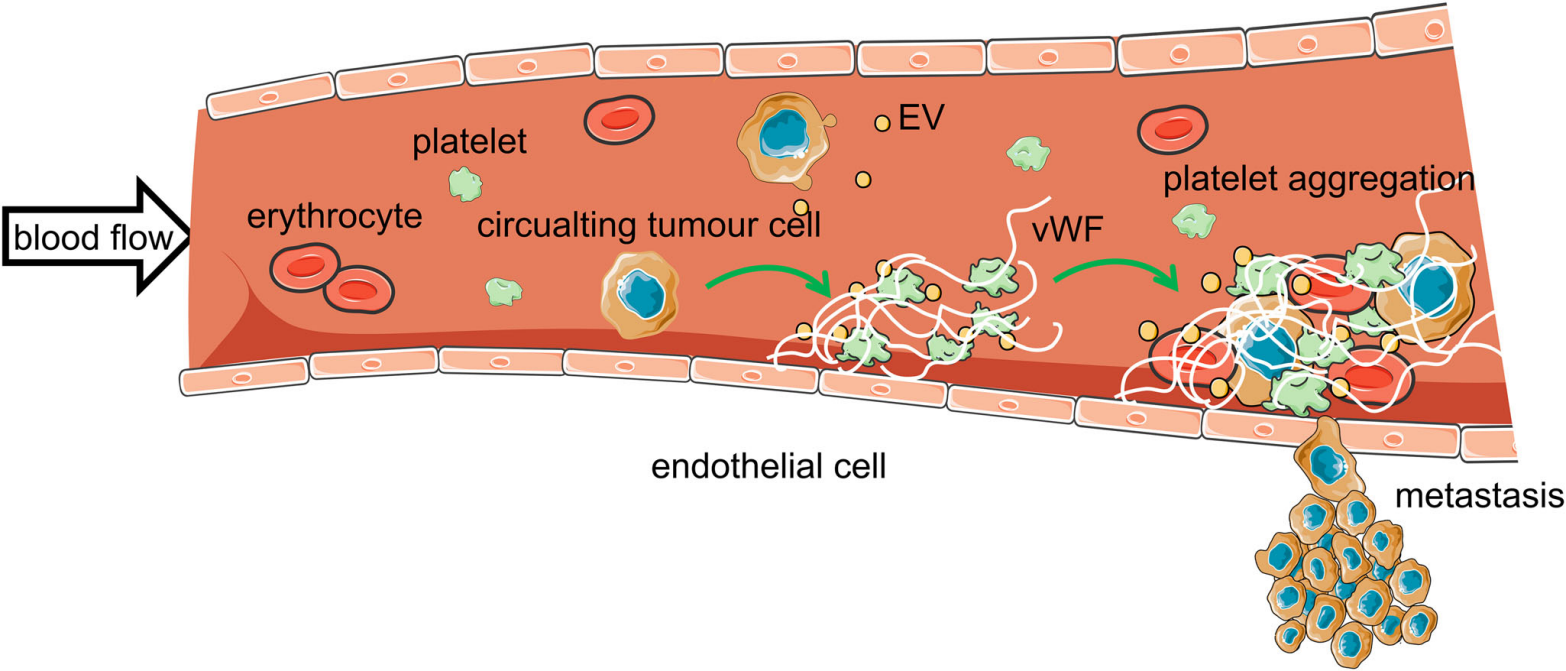
Schematic summary. Schematic summary of the presented data. Under shear flow, vWF released from activated endothelial cells is stretched and binds platelets as well as EVs. EVs can be released from circulating tumour cells or other blood cells such as platelets. In the presence of plasmatic coagulation factors, the binding of TF‐exposing EVs promotes platelet activation and aggregation. Although tumour cells do not directly bind to stretched vWF, platelet aggregation and thrombus formation subsequently entraps erythrocytes and circulating tumour cells, facilitate tumour metastasis. (Schematic elements used from Servier Medical Art: https://smart.servier.com/).

Taken together, our data support a model in which vWF acts as a size‐selective scaffold, preferentially binding small vesicles such as EVs, but not tumour cells directly. Once retained by vWF fibres under shear flow, EVs promote local platelet activation and aggregation, forming thrombus‐like structures capable of capturing circulating tumour cells. These aggregates not only enhance tumour cell viability and migration but also facilitate metastatic colonization. The proposed mechanism is summarized in Figure 9.

## 4 Discussion

The findings presented in this study shed light on the intricate interplay between vWF fibres, EVs and cells. In the past, many molecules and cells have been identified as ligands for vWF indicating that vWF is an important protein within our circulation and involved in a large range of biological processes. Multimeric vWF is considered as key player in coagulation but may also contribute to other endothelial functions such as angiogenesis (Mobayen et al. 2023) or immune cell recruitment (Petri et al. 2010). In the present study, we investigated the balance between the shear force‐dependent opening of the A1 domain promoting adhesion and the shear force‐dependent drag force counteracting adhesion. In line with previous studies, opening of the A1 domain started in our microfluidic experiments (vWF‐coated surfaces) at a shear stress of around 20 dyn/cm^2^ (Huck et al. 2014; Kalagara et al. 2018; Schneider et al. 1997). Less shear stress (5 dyn/cm^2^) was required to elongate endothelial cell‐derived vWF (HUVEC released vWF). These differences are explained by the high molecular weight of endothelial vWF and an increased stress susceptibility upon cell release (Huck et al. 2014). To measure the impact of the drag force in a standardized way, we utilized PE particles with a size range from 0.5 to 10 µm. We found that smaller particles (<4 µm) have a higher ability to interact with vWF coated surfaces. Larger particles (≥ 4 µm) in the size of cells were unable to attach, suggesting that the drag force exceeded the adhesion force. Similar results were obtained in our calculations and also in experiments with whole melanoma cells and melanoma cells derived EVs. Our data indicate that particle binding to vWF under flow depends strongly on particle size. However, the inability of the 10 µm particles to bind may not only be caused by their size, but also by their comparatively low negative surface charge (zeta potential). In future studies, we aim to extend our theoretical calculations by advanced computer simulations that incorporate additional parameters, including particle margination, particle deformation, vWF fibre flexibility, and multivalent interactions, all of which are likely to influence the precise adhesion threshold in the biological system under flow (Cooley et al. 2018).

In comparison to platelets, EVs bound less efficiently, although according to our calculation and previous work (Pino et al. 2014), their size would favour an even stronger binding ability. Our theoretical calculation considers simple sphere‐shaped particles that cannot deform under shear stress. Platelets are however, soft structures with a comparable low elastic modulus (Du Plooy et al. 2013; Liu et al. 2019) promoting their flattening under shear stress. Flattening increases the adhesion area of platelets and thus their net ability to adhere to a vWF‐coated surface. EVs are much smaller and their elastic modulus was found to be relatively high (Calò et al. 2014). In comparison to platelets, it can therefore be assumed that the ability of EVs to flatten under flow is low and that in turn the adhesion area is not affected by shear stress. Not only platelets but also larger cells such as leukocytes or tumour cells may have the ability to deform under flow, which may increase the area of adhesion. Previous studies already indicate that tumour cells with a lower elastic modulus are more metastatic suggesting also an increased ability to attach to the vascular wall (Wang et al. 2023).

The formation of vWF‐platelet strings is a locally restricted process that enables the response of a defined vascular area to endothelial cell activation or injury. The binding of platelets to vWF via GPIbα is a transient process and contraction of the vWF fibre leads to the dissociation of the platelets (Auton et al. 2010). Therefore, the formation of stable aggregates requires an additional activation of platelets and a stabilization of the vWF‐platelet conglomerate through fibrin. Thrombin, the downstream product of TF, is a key enzyme facilitating the activation of platelets through protease‐activated receptor signaling and the conversion of fibrinogen into fibrin (Camerer et al. 2004). In addition, thrombin generation requires assembly of coagulation factors on anionic phospholipid‐rich surfaces, which may be provided not only by TF‐positive EVs but also by activated platelets. Previous work has shown that, under high shear and in the presence of vWF, platelets express P‐selectin and bind annexin V, consistent with the acquisition of a procoagulant phenotype (Gao et al. 2008). Moreover, protease‐activated receptor signaling in platelets triggers the activation of GPIIb/IIIa, which binds to the RGD motif of vWF (Camerer et al. 2004; Mojzisch and Brehm 2021) and thereby promotes platelet aggregation. After its local production, thrombin is rapidly diluted in the circulation and due to a relatively short life‐time eliminated within seconds (Rühl et al. 2012). Here, we found that EVs bind to vWF fibres in close proximity to platelets increasing the likelihood of a locally restricted platelet aggregation. Additionally, also the low abundance of TF‐positive EVs as shown by imaging flow cytometry may support a localized effect. Thus, our data support a model in which EV‐associated TF and platelets cooperate to promote local coagulation and stabilization of the vWF‐platelet conglomerate. The prometastatic role of platelets and TF‐induced coagulation is well documented and the direct physical interaction of platelets and tumour cells is known to protect the tumour cells against the attack of the immune system and to support metastasis (Garcia‐Leon et al. 2024; Lucotti et al. 2025; Rack et al. 2017). This is consistent with our data, showing the improved migration of B16F10 cells through collagen gels in the presence of EV‐vWF‐platelet conglomerates, as well as increased metastatic burden in vivo, although vWF‐dependent EV binding is likely to represent only one component of the broader prometastatic activity of EVs (Che et al. 2017; Li et al. 2024; Wang, Sun, et al. 2022).

Our experiments indicate that tumour cell‐derived EVs bound to vWF via HS, which is ubiquitously expressed by mammalian cells, suggesting that vWF fibres might also capture EVs from other cell types. In addition, other membrane components may contribute to this interaction, as phosphatidylserine has also been reported to bind vWF (Nicolay et al. 2018). Given that EVs are produced by almost all types of cells, their levels in the blood circulation are high. While most EVs originate from platelets, those derived from leukocytes (Liu et al. 2026), red blood cells, and circulating tumour cells also contribute to blood coagulation (Alberro et al. 2021). Interestingly, previous studies discovered that EVs derived from sickle red blood cells or neutrophils promote the release of vWF from the endothelium to enhance thrombotic processes and future experiments will show whether the binding of EVs to vWF fibres contribute to thrombosis in such diseases (An et al. 2023; Liu et al. 2022; Yang et al. 2020).

As aforementioned, vWF elongation takes place at a relatively broad shear stress range (∼5 dyn/cm^2^ endothelial cell released vWF, ∼20 dyn/cm^2^ vWF coated surfaces) suggesting a contribution of the EV‐vWF interaction to different pathophysiological conditions. Moreover, it has been reported that the amount of TF^+^‐EVs in the blood increases under various disease conditions (Hisada et al. 2022; Sachetto et al. 2023). Therefore, we hypothesize that the binding of TF^+^‐EVs to vWF is not limited to cancer‐associated thrombosis, but is a general mechanism contributing to vWF‐mediated platelet aggregation and coagulation, for example, to seal vascular injuries or to contribute to hypercoagulation in disease. EVs were postulated as biomarkers predicting disease progression and severity. For example, clinical observations suggested that vWF together with EVs from gastric cancer cells are associated with cancer aggression and poor clinical outcomes for patients (Cai et al. 2023). Also, the exposure of TF by EVs was shown to be linked to lung metastasis in a murine tumour model (Lucotti et al. 2025).

Here, we found that loss of HS markedly reduced EV adhesion to vWF fibres, suggesting that blocking the HS‐binding interface on vWF by HS mimetics may represent a potential therapeutic approach to inhibit EV‐driven thrombosis and metastasis. Moreover, our data demonstrate that vWF fibres function as vascular traps for nanoparticles within tumour vessels and possibly also within pre‐metastatic niches, which have previously been shown to display enhanced vWF fibre formation (Goertz et al. 2016). This aligns with recent work highlighting how blood flow and particle size govern vascular targeting efficiency (Lee et al. 2025). In this context, vWF fibre networks may represent a physiologically occurring scaffold that enhances the localization of circulating drug carriers at sites of endothelial activation.

## 5 Conclusion

In conclusion, our study shows that the binding of EVs to stretched vWF could activate platelets and trigger platelet aggregation. These thrombus‐like aggregates can trap circulating tumour cells and significantly promote metastatic outgrowth. By elucidating the molecular and biophysical mechanisms underlying EV‐vWF interactions, our study may support the future development of therapeutic approaches targeting EV‐mediated thrombosis in cancer patients and in other diseases characterized by hypercoagulation. Additionally, our findings underscore the clinical relevance of EVs and vWF as potential biomarkers for assessing thrombotic risk and monitoring disease progression.

## Author Contributions

**Yuanyuan Wang**: conceptualization, investigation, funding acquisition, writing – original draft, writing – review and editing, visualization, validation, methodology, software, project administration, formal analysis, data curation. **Xiaobo Liu**: writing – review and editing, methodology, investigation, conceptualization, validation, software, data curation. **Pascal Nakielski**: methodology, writing – review and editing. **Katrin Nekipelov**: methodology, writing – review and editing. **Amanda Salviano–Silva**: methodology, writing – review and editing, data curation, validation. **Alper Topuz**: methodology, software, writing – review and editing. **Alexander T. Bauer**: writing – review and editing, investigation. **Julian Kött**: writing – review and editing, resources. **Jannis Akrivakis**: methodology, writing – review and editing. **Santra Brenna**: methodology, writing – review and editing, validation. **Tomasz Downar**: methodology, writing – review and editing. **Neus Feliu**: writing – review and editing. **Gerd Bendas**: writing – review and editing. **Berta Puig**: writing – review and editing. **Stefan W. Schneider**: writing – review and editing, conceptualization, funding acquisition. **Dmitry A. Fedosov**: writing – review and editing. **Christian Gorzelanny**: writing – review and editing, conceptualization, investigation, funding acquisition, supervision, project administration, data curation.

## Funding

This study was supported by research funding from the Mildred Scheel Cancer Career Center HaTriCS4 at University Medical Center Hamburg‐Eppendorf; the German Research Foundation within priority program 2416 “CodeChi” (GO 2528/10‐1), GRK 2873 ‘*Tools and Drugs of the Future*’, CRC1700 (Project No 530990199); and the Hamburg Pro Exzellenzia Plus Scholarship Program.

## Ethics Statement

All animal experiments were approved by the governmental animal care authorities (‛Behörde für Justiz und Verbraucherschutz, Hamburg’ project N033/2022). Blood samples from human volunteers were collected after written informed consent and in accordance with the guidelines of the Ärztekammer Hamburg. The study was approved by the Ethics Committee of the Ärztekammer Hamburg (MC‐395/17).

## Consent

All authors have agreed to publish this manuscript.

## Conflicts of Interest

The authors declare no conflicts of interest.

## Supporting information




**Supporting Information**: jev270349‐sup‐0001‐SuppMat.pdf

**Supporting Information**: jev270349‐sup‐0002‐VideoS1.mp4

**Supporting Information**: jev270349‐sup‐0003‐VideoS2.mp4

## Data Availability

All data generated or analysed during this study are included in this published article and the Supplementary materials. For original data, please contact corresponding author.
