# Supplementary material for: Shear‐Activated von Willebrand Factor Captures Extracellular Vesicles to Promote Platelet Activation and Metastasis
Source: J Extracell Vesicles. 2026 Aug 9;15(8):e70349. doi: 10.1002/jev2.70349 (PMC13453939; doi:10.1002/jev2.70349)
Supplement: Supplementary file 1 [file JEV2-15-e70349-s002.pdf]

Supplementary materials

Shear-activated von Willebrand factor captures extracellular vesicles to promote platelet activation and metastasis

Running title: Shear-activated vWF captures EVs

Yuanyuan Wang[1,2+], Xiaobo Liu[1+], Pascal Nakielski[3], Katrin Nekipelov[4], Amanda Salviano-Silva[5], Alper Topuz[6], Alexander T. Bauer[1], Julian Kött[1,7], Jannis Akrivakis[1], Santra Brenna[8], Tomasz Downar[9], Neus Feliu[3], Gerd Bendas[4], Berta Puig[8], Stefan W. Schneider[1], Dmitry A. Fedosov[6], Christian Gorzelanny[1]

[1] Department of Dermatology, University Medical Center Hamburg-Eppendorf, Hamburg, Germany.

[2] Mildred Scheel Cancer Career Center HaTriCS4, University Medical Center Hamburg-Eppendorf, Hamburg, Germany.

[3] Fachbereich Physik, CHyN, Universität Hamburg, Hamburg, Germany.

[4] Pharmaceutical Institute, University of Bonn, Bonn, Germany.

[5] Department of Neurosurgery, University Medical Center Hamburg-Eppendorf, Hamburg, Germany.

[6] Institute for Advanced Simulation (IAS-2), Forschungszentrum Jülich, Jülich, Germany.

[7] Fleur Hiege Center for Skin Cancer Research, University Medical Center Hamburg-Eppendorf, Hamburg, Germany.

[8] Neurology Department, Experimental Research in Stroke and Inflammation (ERSI), University Medical Center Hamburg-Eppendorf, Hamburg, Germany.

[9] Experimental Dermatology, Medical Faculty Mannheim, Heidelberg University, Mannheim, Germany.

[+] X.L. and Y.W. contributed equally to this work

Corresponding author:

Christian Gorzelanny, Department of Dermatology, University Medical Center Hamburg-Eppendorf, Martinistrasse 52, 20251, Hamburg, Germany
phone: +49 40 7410 58976
fax: +49 40 7410 52655
e-mail: c.gorzelanny@uke.de

Supplementary materials includes:

Supplementary Materials and Methods

Supplementary Information: calculation of particle binding under shear stress.

Supplementary Figures 1-9

- **Supplement Figure 1:** Flow rate response to shear stress in microfluidic system.
- **Supplement Figure 2:** Binding of heparin coated PE particles to vWF.
- **Supplement Figure 3:** Binding of blood flowing objects to vWF under shear stress.
- **Supplement Figure 4:** Analysis of the HS-vWF Interaction by single molecular force spectroscopy.
- **Supplement Figure 5:** Binding of EVs to vWF in the absence of platelets.
- **Supplement Figure 6:** Binding of melanoma cell-derived EVs to stretched vWF mutants.
- **Supplement Figure 7:** TF-EV promotes fibrinogen deposition on vWF-bound EV–platelet aggregates under flow.
- **Supplement Figure 8:** Binding of EVs from different different TF-expressing cell lines to vWF.
- **Supplement Figure 9:** EV promoted lung metastasis in ADAMTS13−/− mice.

Supplementary Videos 1-2

- **Video S1:** Platelet binding to vWF fibres under flow.
- **Video S2:** Co-binding of EVs and platelets enhances platelet activation under flow.

Supplementary References

Supplementary Materials and Methods

1. Cell lines and culture conditions

Human melanoma cell lines MV3, Mewo, and A2058 were cultured in RPMI-1640 medium (Sigma-Aldrich). The mouse melanoma cell line B16F10 was cultured in DMEM medium (Sigma-Aldrich). Both media were supplemented with 10% fetal bovine serum (FBS), 1% L-glutamine (Biochrom), and 1% penicillin/streptomycin (Biochrom).

Human umbilical vein endothelial cells (HUVECs) were isolated from human umbilical cords. The umbilical vein was cannulated and perfused with PBS to remove blood. Collagenase type I (0.1% w/v in PBS) was infused into the vein and incubated for 15-20 minutes at 37°C to digest the endothelial cells. The vein was then gently massaged to dislodge the cells. Isolated HUVECs were seeded into flasks pre-coated with 0.1% gelatin and cultured in Endothelial Cell Growth Medium-2 (EGM-2) (Sigma-Aldrich) supplemented with the provided growth factors, 10% FBS, and 1% penicillin/streptomycin. Only cells at passages 2-5 were used for experiments.

All cells were incubated in a humidified atmosphere at 37°C with 5% CO_2 until they reached approximately 90% confluency.

2. HS coating of fluorescent polystyrene particles

Fluorescent polystyrene (PE) particles (0.5 µm PFP-0562, 1.8 µm PFH-2052, 4.0 µm PFP-4070, 10.0 µm PFP-10052 from Kisker Biotech) were incubated with 1 mg/mL streptavidin in 0.1 M phosphate buffer (pH 7.4). The mixture was vortexed and incubated overnight at 4°C. After incubation, the suspension was centrifuged at 3000 × g for 15 minutes, and the supernatant was carefully removed. The pellet was washed with 4 mL isotonic buffered saline (IBS), centrifuged again, and the supernatant was removed. The pellet was then resuspended in IBS and mixed well to obtain a 5% w/v streptavidin-coated particle suspension.

Subsequently, 0.2 mL of the streptavidin-coated particle suspension was incubated with 2.0 mL of 100 µg/mL Biotin-Heparin (Sigma Aldrich) in 0.1 M sodium phosphate buffer (pH 5.5). The mixture was vortexed and incubated overnight at 4°C. After incubation, the suspension was centrifuged at 3000 × g for 10 minutes, and the pellet was washed three times with 4 mL of 0.1 M sodium phosphate buffer (pH 5.5). After each wash, the supernatant was carefully removed. Finally, the pellet was resuspended in 0.1 M sodium phosphate buffer (pH 5.5) to obtain a 1% w/v suspension.

3. EV isolation

EVs were isolated from conditioned medium using a differential ultracentrifugation workflow. Cell were cultured in T75 flasks in 12 mL serum-free medium (Opti-MEM, Thermo Fischer) for 24 hours. The collected medium was sequentially centrifuged at 500 × g for 10 min, 2,000 × g for 15 min at 4°C, and 10,000 × g for 30 min to remove cells, debris, and larger particles. The resulting supernatant was then ultracentrifuged at 100,000 × g (SW40Ti rotor) for 70 min at 4°C. After carefully discarding the supernatant, the EV-containing pellet was resuspended in sterile, filtered phosphate-buffered saline (PBS) for downstream analyses.

4. Fluorescence labelling of EVs

After the isolation from cell culture medium, EVs were labelled with the lipophilic tracer DiD (1,1′-Dioctadecyl-3,3,3′,3′-tetramethylindocarbocyaninperchlorat, Thermo Fischer). The labelling was performed on ice and in the dark for 10 minutes. To remove any unincorporated dye, the labelled EVs were washed with PBS and centrifuged at 100,000 × g (SW40Ti rotor) at 4°C for 70 minutes. The resulting pellet containing the labelled EVs was resuspended in PBS and used for the experimental procedures.

5. Single molecule force spectroscopy

Single molecule force spectroscopy (SMFS) measurements were performed with an atomic force microscope (AFM) (NanoWizard, JPK) as previously reported.[1] AFM tips (CS38/No Al,

microMasch) and glass slides (Thermo Fisher Scientific) were functionalized with a polyethylene glycol (PEG) linker (Nanocs) and streptavidin was covalently attached to the linker through amino functionalization. Biotinylated HS (Sigma-Aldrich) was coupled to the streptavidin functionalized surfaces. Prior to the SMFS experiment, recombinant vWF in PBS (1µg/ml) was added to the glass slide and force distance cycles were immediately initiated. Rupture forces were recorded at different loading rates. At least 500 force curves per loading rate have been analyzed. The off rate and the bond length of the interaction between vWF and HS were determined as previously reported.[2]

6. Surface acoustic wave biosensor measurements

Surface acoustic wave (SAW) measurements were conducted using a Sam®5 Blue acoustic biosensor (SAW Instruments GmbH) to analyze binding interactions between vWF and EVs. A model membrane containing 20 mol% 1,2-Di-(9Z-octadecenoyl)-sn-glycero-3-[(N-(5-amino-1-carboxypentyl)iminodiacetic acid)succinyl nickel salt] (DGS-NTA(Ni)) and 80 mol% dipalmitoylphosphatidylcholine (DPPC, Sigma-Aldrich) was prepared on the sensor surface as previously described.[3] Baseline equilibration was achieved under buffer flow (40 µL/min). For vWF-EV interaction studies, 4.9 µg his-tagged vWF was injected at a flow rate of 20 µL/min to enable binding to the DGS-NTA(Ni) lipids in the membrane. The measurements were run at room temperature with a continuous buffer flow of 40 µL/min. After each injection of EVs, a rinse injection of 2 M NaCl solution was carried out in order to remove the EVs from the protein. A dilution series of B16F10 wild-type (WT) or Ext1−/− EVs (1:200 to 1:25 in running buffer: 1 mM $CaCl_2$, 1 mM $MgCl_2$, 0.5 mM $MnCl_2$) was then injected, and real-time phase shifts were recorded.

7. Mouse experiments

All animal experiments were approved by the governmental animal care authorities ("Behörde für Justiz und Verbraucherschutz, Hamburg" project N033/2020). C57BL/6J mice and vWF deficient mice (Jackson Laboratory) were maintained under specific pathogen free conditions.

Mice were randomized to experimental groups; data acquisition and analysis were performed blinded to group allocation. The only pre-specified exclusion criterion was tail-vein injection failure, and excluded animals were recorded as previously described.[1].

Nanocapsule accumulation

Melanoma cells were injected intradermally (i.d.) into 8-12 week-old wild-type (WT) and von Willebrand factor knockout (vWF−/−) mice. After two weeks, HS-coated fluorescent nanocapsules (diameter: 157 ± 53 nm) were administered intravenously (i.v.). Twenty-four hours after nanocapsule administration, the mice were sacrificed. Tumour blood vessels containing accumulated nanocapsules were embedded for cryosectioning and subsequently analysed by immunofluorescence analysis.

EV-assisted metastasis

Melanoma cells (5 × 10^5) alone or co-injected with WT or TF−/− EVs (1 × 10^9) were delivered intravenously (i.v.) into 8–12-week-old WT and ADAMTS13-deficient (ADAMTS13−/−) mice. Two weeks post-injection, mice were euthanized, lungs were embedded for cryosectioning, and pulmonary metastases were analyzed (number and area).

8. EVs characterization

Western blot

The total protein content in EVs or cell lysates were determined using the Micro BCA Protein assay kit (Thermo Scientific), following the manufacturer's instructions. After quantification, the samples were denatured at 70°C for 10 minutes with NuPage LDS Sample Buffer (Invitrogen) and NuPage sample reducing agent (Invitrogen). Equal protein amounts were loaded onto NuPage 10% Bis-Tris gels (Invitrogen) and separated by electrophoresis. Proteins were transferred to a Transblot Turbo nitrocellulose membrane using wet-blotting. The membranes were then blocked for 1 hour and incubated with primary antibodies overnight at 4°C on a shaking platform. The following primary antibodies were used: rabbit antibody against Alix

(1:400; #2171 CST, Millipore), rabbit monoclonal against CD81 (1:1000; #10037, Cell Signalling), mouse antibody against flotillin-1 (1:1000; #610820, BD Biosciences), mouse antibody against GM130 (1:1000; #610822, BD Biosciences). After washing with TBST (Tris-buffered saline with Tween), the membranes were incubated for 1 hour with the respective HRP-conjugated secondary antibodies (1:1000, Cell Signaling) and subsequently washed six times with TBST. The protein bands were visualized using a chemiluminescence imaging system (azure biosystems, Dublin, USA).

Nanoparticle tracking analysis

EVs were subjected to Nanoparticle tracking analysis (NTA) for characterization, following a previously described protocol.[4] Briefly, 1 µL of the EVs suspension was diluted at 1:5000 in PBS and 500 µL of the diluted sample was loaded into the sample chamber of an LM10 unit (Nanosight). Ten videos of ten seconds were taken for each sample with a frame rate of 30 frames/sec. The movement of particles was subsequently analyzed using the NTA 3.0 software (Nanosight). The detection threshold was set to six and the screen gain to two.

Flow cytometry

The NovoCyte Quanteon flow cytometer was used to characterize EVs. Fluorescent polystyrene particles from Kisker Biotech with diameters of 0.1 µm (PFP-0245), 0.8 µm (PFP-0862), 1.8 µm (PFH-2052), and 4.0 µm (PFP-4070) served as size references to create a dimensional gate for EVs. Data analysis was performed using the Flowing Software (version 2.5.1).

Transmission electron microscopy

Transmission electron microscopy (TEM) was utilized as previously described.[5] Briefly, EV samples were prepared for negative staining. A carbon-coated formvar grid (2 nm carbon) was placed on a 20 µL drop of the EV sample, allowing it to adsorb for 10 seconds. The grid was subsequently washed three times with water drops and stained with a 3% uranyl acetate

solution. After drying, the micrographs were captured using a transmission electron microscope (JEM 1400; JEOL Ltd) equipped with a bottom-mounted high-sensitivity 4K CMOS camera (TemCam F416; TVIPS).

Stimulated emission depletion microscopy

EVs were collected and labelled with DiD as described earlier. The labelled EV pellet were resuspended in 15 µL Fluoromount G (Southern Biotech). EVs were deposited on a glass slide and covered with a coverslip. Stimulated emission depletion (STED) microscopy experiments were conducted under similar conditions as previous studies.[1] Briefly, imaging was conducted in sequential line scanning mode with an Abberior STED expert line microscope. Excitation was achieved with a pulsed 640 nm laser, while depletion was performed using a near-infrared pulsed 775 nm laser. The dwell time for image capture was 3 ms, with a voxel size of 20 × 20 × 150 nm. Images were acquired in time-gating mode with a gating width of 8 ns and a delay of 781 ps. Data analysis was carried out by ImageJ software (version 1.54i).

9. Platelets and erythrocytes isolation

Human citrated blood was collected and left at RT for 30min, followed by centrifugation at 150 × g for 15 minutes without break, yielding three distinct phases: platelet-rich plasma with plasmatic proteins and platelets, the "buffy coat" interphase containing leukocytes, and the erythrocyte phase primarily composed of red blood cells. The platelet-rich plasma was gently transferred to a 15 mL conical bottom tube and mixed in a 1:1 ratio with washing buffer (136 mM NaCl, 2.7 mM KCl, 0.54 mM NaH2PO4 × H2O, 5 mM HEPES, 2.8 mM dextrose, pH 6.5), which included 1 U/ml of the platelet inhibitor apyrase. After washing, platelets (shown in green in Figures) were stained with CellTrace™ Calcein Red-Orange (ThermoFisher) for 15 minutes. Subsequently, the platelet was washed again and centrifuged at 1200 × g without break for 15 minutes. The supernatant was discarded, and the platelet pellet was resuspended in resuspension buffer (136 mM NaCl, 2.7 mM KCl, 0.54 mM NaH2PO4 × H2O, 11.9 mM NaHCO3, 5.6 mM glucose, 5% w/v bovine serum albumin (BSA), pH7.4). The erythrocyte

pellet obtained from the citrated blood was transferred to a 50 mL tube and washed with an equal amount of HEPES-buffered Ringer's solution (136 mM NaCl, 2.7 mM KCl, 1mM $CaCl_2$, 1 mM $MgCl_2$, 10 mM HEPES, 5 mM glucose) for three times. After each washing step, erythrocytes were centrifuged at 800 × g for 10 minutes. The supernatant was removed to obtain a purified pellet of washed erythrocytes.

10. Microfluidic experiment

Binding of EVs or particles to recombinant vWF

BIOFLUX 200 48-WELL plates were coated with 20 µg/mL of recombinant wild-type vWF (WT vWF) or recombinant vWF mutants lacking the A1 or A2 domain (ΔA1 or ΔA2 vWF) for 1 hour at room temperature. Tumour cell-derived EVs or HS-coated PE particles (0.5 µm PFP-0562, 1.8 µm PFH-2052, 4.0 µm PFP-4070, 10.0 µm PFP-10052) were perfused through the BIOFLUX200 system with or without platelets or erythrocytes. Applied shear forces ranging from 10 to 80 dyn/cm².

Adhesion of particles, platelets (CellTrace™ calcein red-labeled, ThermoFisher, shown in green in Figures), tumour cells (CellTrace™ calcein green-labeled, ThermoFisher, shown in blue in Figures) and EVs (DiD-labeled, shown in black in Figures) to vWF fibres was visualized by fluorescence microscopy (Observer Z.1, Zeiss). The resulting images were analyzed with the ImageJ software (version 1.54i) to quantify adhesion and binding events. Platelet coverage and aggregation were quantified based on fluorescence intensity.

EVs binding to endothelial vWF

HUVECs were cultured to confluence on fibronectin-coated µ-Slide I Luer (Ibidi) using Leibovitz's L-15 Medium (Gibco) at 37°C. To stimulate the release of vWF, HUVECs were treated with 100 µM histamine under flow conditions. DiD-labeled EVs (shown in red in Figures) were perfused through the BIOFLUX200 system at a shear stress of 5 dyn/cm².

11. Immunofluorescence staining of cells

Cells were fixed in 4% paraformaldehyde for 10 minutes, washed with PBS and blocked with 10% goat serum. The primary tissue factor antibody (Abcam, ab189483, 1:50) and secondary antibody: Alexa 555-conjugated goat anti-rabbit (IgG; Thermo Fisher Scientific, 1:500) were used for staining. Samples were imaged by fluorescence microscopy (Observer z.1, Zeiss). Image analysis was performed using the Image J software (version 1.52).

12. Flow cytometry for cells

Cells were collected and incubated with a TF antibody (rabbit anti human or mouse IgG, Gene Tex 1:100) for 1 hour on ice. After washing with PBS, TF antibodies were further labeled with Alexa Fluor 555 goat anti-rabbit IgG secondary antibodies (Thermo Fischer Scientific, 1:500) for 30 minutes on ice. Fluorescence signals were evaluated by flow cytometry (BD FACSCanto II, Biosciences). Data were analyzed by the Flowing Software (version 2.5.1).

13. Survival and migration of tumour cells

Tumour cell survival and migration were assessed using a basement membrane-like collagen matrix gel in µ-Slide 15 Well 3D ibiTreat chambers (ibidi). The gel was prepared using 100 µg/mL collagen I (Sigma-Aldrich) and precisely pipetted (10 µL) into the inner wells of the µ-Slide to achieve a meniscus-free, flat surface. Gelation was performed in a humidity chamber within an incubator for 30–60 minutes.

After gelation, 10,000 B16F10 cells, with or without platelets, were seeded onto the slides and incubated for 48 hours. The experiment was stopped by fixation of the gel with 4% paraformaldehyde. Gels were frozen and cut into 10 µm slices with a cryostat (CM3050S, Leica Biosystems). Nuclei were stained with DAPI; platelets were labeled with an antibody directed against thrombospondin 1 (Epredia MS-421-P). Fluorescence images were captured with a fluorescence microscope (Observer z.1, Zeiss). Aspect ratios were calculated as major/minor axis lengths using Image J (version 1.52).

14. Genetic engineering of melanoma cells

Tissue factor knockdown (shRNA). F3 -Mouse shRNA lentiviral particles (ORIGENE, 134DA7D; 4 unique target-specific shRNA and 1 scramble control) were used to transduce B16F10 cells as described previously.[1] Transduced cells were selected with puromycin (dose optimized per cell line) to generate stable polyclonal pools. Knockdown efficiency was verified by RT-qPCR and IF.

Tissue factor knockout (CRISPR/Cas9). Single-guide RNAs (sgRNAs) targeting murine F3 were designed with CRISPOR (http://crispor.tefor.net/) and cloned into pLenti CMV Blast mKate2. The target sequences were: gRNA1 (+) 5′-GTCCTCTCTGTCCCACGG**AGG**-3′; gRNA2 (+) 5′-TCGTGAAGGATGTGACCT**GGG**-3′ (PAM underlined). Lentiviral particles for each sgRNA were produced separately, titer-matched, mixed 1:1, and used to transduce B16F10 cells as described previously.[1] Transduced cells were selected with puromycin, single-cell cloned by limiting dilution, and screened using the Alt-R Genome Editing Detection Kit, followed by Sanger sequencing of PCR amplicons flanking the target site (primers: 5’-TGCCCAGCAGAGCTAACCTGGTTAC-3’ and 5’-GATCTAATTAATGGCTAATAGGGGGTCATG-3’). Cells transduced with non targeting sgRNA served as controls.

15. Zeta potential

The ζ-potential of the particles was measured using a Malvern Zetasizer Nano series instrument equipped with a Zetasizer Dip Cell Kit. Samples were diluted in deionized water and loaded into the zeta dip cell, avoiding the formation of air bubbles. Water was used as the dispersant, with a refractive index of 1.330, viscosity of 0.8872 cP, and dielectric constant of 78.5. Measurements were performed at 25 °C. For each sample, minimum 12 zeta runs were recorded with a measurement position of 5.00 mm. The ζ-potential was calculated from the electrophoretic mobility using the instrument software and reported as the mean ± deviation of repeated measurements as previously reported[6].

16. Imaging flow cytometry

EV populations carrying tetraspanins (CD81 and CD9) and TF were quantified by Imaging Flow Cytometry (IFCM). 5 µL of EVs were mixed with 3 µL of IFCM buffer (filtered PBS containing 2% exosome-depleted FBS [Invitrogen, cat. no. A2720801[) and stained with PE-cojugated goat anti-mouse TF (R&D Systems, cat.# FAB3178P, 2uL), AlexaFluor647-conjugated hamster anti-mouse CD81 (Bio-Rad, clone Eat2, 0.1 µg) and AlexaFluor647-conjugated rat anti-mouse CD9 (Biolegend, clone MZ3, 0.5 µg). The EV-antibody solutions were incubated in the dark for 45 minutes at room temperature, and washed twice with 500 µL of IFCM buffer using a 300 kDa filter (Nanosep, 4,000 g for 7 minutes, 4°C). Stained EVs were resuspended from the filter in 30 µL of IFCM buffer. IFCM negative controls included EVs without antibodies, as well as full staining with lysed EVs (0.5% of NP40 for 30 min). Data was acquired on ImageStreamX Mark II Imaging Flow Cytometer (Amnis, Luminex Corporation), at 60× magnification, with low flow rate and beads removal. Fluorescent signals were detected for PE in channel 3 (560–595 nm filter; laser voltage: 100 mW) and AF647 in channel 11 (642 nm filter; laser voltage: 100 mW). Non-swarmed values were analyzed using IDEAS software version 6.2 (Amnis, Luminex Corporation).

Supplementary Information

To interpret our experimental data shown in Figure 2, we calculated the binding of PE-particles to vWF at different shear stress conditions as illustrated schematically in S-Figure 3. We calculated the drag force (F_{drag}) pulling at the particle by Stoke's law:

$$F_{drag} = 6\pi R\mu\gamma(R + s_0) \qquad (1)$$

where μ is the viscosity of the perfusing medium (0.01 dyn s/cm²), γ the shear stress and s_0 the separation of the particle from the surface. R is the radius of the particle. We considered the drag force only of particles that are very close to the vascular wall where $R \gg s_0$.Equation 1 is therefore simplified to:

$$F_{drag} = 6\pi R^2\mu\gamma \qquad (2)$$

To estimate the adhesion force (F_{ad}), we used the previous approach of Jiang and co-workers, which have calculate the interaction force of GPIbalpha and the A1 domain of vWF.[7] To estimate the interaction force of HS and vWF, we performed single molecular force spectroscopy using an atomic force microscope (S-Figure 4). We evaluated our data assuming that the rupture of the HS-vWF interaction is related to the applied loading rate as previously described[8]. We found a bond length of 0.399 ± 0.096 nm and an off-rate of 1.6 ± 0.8 1/s. Our data are in good agreement with previous measurements of the interaction of different vWF A1 domain constructs with GPIbα, which documented a bond length range from 0.44 to 1.4 nm and off-rates between 3.27 and 10.8 1/s.[9] Therefore, we assumed that the binding of HS-coated particles is steered by the same positively charged amino acids of the A1 domain. The interaction force between the A1 domain and HS depends strongly on their separation, s, as indicated by equation 3:

$$F_{A1-HS}(s) = \sum_{i,j} -\frac{q_i q_j}{4\pi\varepsilon_0\varepsilon}\frac{e^{\kappa r}}{(1+\kappa r)}\frac{(\kappa s+1)e^{-\kappa s}}{s^2} + \frac{6C_{vdw}}{s^7} \qquad (3)$$

where the charges, q_i, of the positively charged aminoacids of the A1 domain have been listed in the publication of Jiang et al.[7] The charges of HS, q_j, were set to 1. ε and ε_0 are the relative and the vacuum dielectric constants, respectively. The radii, r, of the hydrated charged groups were estimated to be in the range of 3.5 Å and, at a monovalent salt concentration of 150mM, the debye length $1/\kappa$ was calculated to be 7.7 Å.

We added to the previously published equation[10] a term accounting for weak van der Waals interactions promoting particle adhesion at short distances and independent of the electrostatic interaction. The corresponding constant C_{vdw} was set to -6.66×10^{-83} J m^6.

Further, we assumed that multiple A1 domains can interact with distinct binding partners at the particle surface. The distance, d, of the binding partners was estimated to be 500nm and the resulting separation s' of the multiple binding sites was calculated using equation 4:

$$s' = \cos\left(\tan^{-1}\frac{d}{R+s_0}\right)\left(\sqrt{(R+s_0)^2+d^2}-R\right) \tag{4}$$

where s_0 accounts for the shortest distance between the adhering particle and the surface as indicated in the schematic drawing shown in S-Figure 3A. To estimate the adhesion force (F_{ad}) in three dimensions, we extended equation 2:

$$F_{ad}(s) = \left[\frac{2\pi R}{d}\sum_{l} F_{A1-HS}(s')\right] + F_{A1-HS}(s_0) \tag{5}$$

We aimed to calculate the number of particles bound to a vWF-coated surface at different shear stress conditions. Increasing shear stress promotes the elongation of vWF and opening of the A1 domain. The percentage of open A1 domains at defined shear stress was quantified by measuring the adhesion of platelets to vWF. At a wall shear stress of 4 dyn/cm², 1.5% of the A1 domains were open, at 8 dyn/cm² 5.2%, at 16 dyn/cm² 12.5%, at 32 dyn/cm² 52%, at

64 dyn/cm² 80%, and at 128 dyn/cm² 98%. Additionally, increasing shear stress is directly linked to an increased flow rate and therefore to an increased amount of particles flowing over the adhesive surface. The number of particles, *n*, accumulating over time in the field of view (FoV) of the microscope is linked to the wall shear rate $\dot{\gamma}_w$:

$$n(t) = \frac{w\dot{\gamma}_w h^2}{2}\, t c_{total} \qquad (6)$$

where *w* is the width of the FoV, *h* is the height of the FoV, *t* is the time of the experiment and c_{total} the total particle concentration (3x10^{12} particels/ml). The wall shear stress is linked to $\dot{\gamma}_w$ through the viscosity, μ.To estimate the number of particles close enough to the vWF-coated surface to be trapped, we defined a critical separation, s_{crit}, of the flowing particles from the surface. As illustrated in S-Figure 3B, s_{crit} defines the separation when F_{drag} is equal to F_{ad}. To link s_{crit} to the number of bound particles, n_{bound}, we adjusted equation 6:

$$n_{bound}(t) = \frac{w\dot{\gamma}_w s_{crit}^2}{2}\, t c_{total} \qquad (7)$$

Note that in our calculation we have ignored the potential effect of particle margination toward the wall.[11,12] We expect that particle margination will not significantly change the results, since particle migration toward the wall is slow and is likely not very pronounced in relatively short microfluidic channels used in the experiments.

Supplementary Figures

Figure S1, related to Figure 1

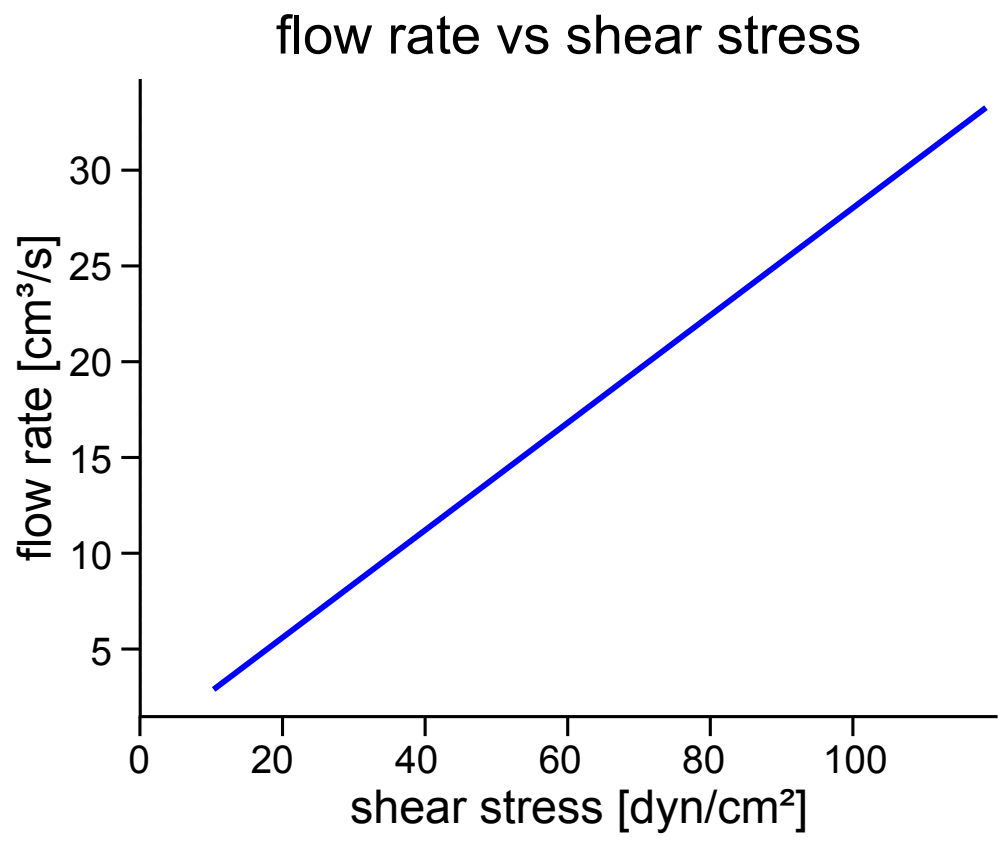

Supplement Figure 1. Flow rate response to shear stress in microfluidic system. Shown is the relationship between the flow rate and the shear stress , demonstrating monotonic increase from 0 to 30 cm^3/s across the tested range (0-80 dyn/cm^2).

Figure S2, related to Figure 2

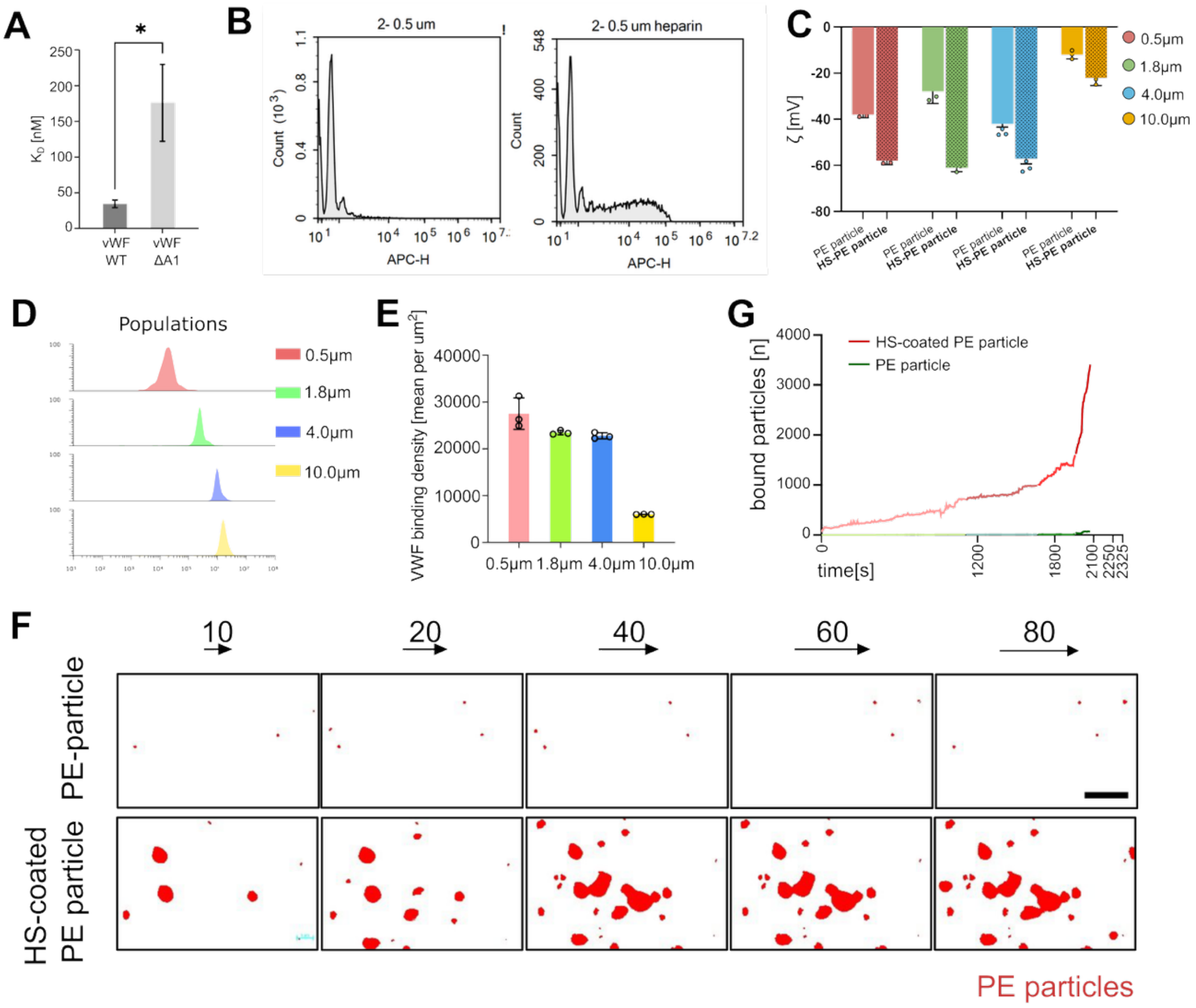

Supplement Figure 2. Binding of heparin coated PE particles to vWF. (**A**) Dissociation contant (K_D) of the interaction between heparin with WT or ΔA1 vWF (n=3); lower K_D indicates higher affinity. (**B**) Heparin coating efficiency on PE-particles was measured by flow cytometry. The rightward shift of the peak indicates successful heparin conjugation to the particle surface. (**C**) Zeta potential of PE particles of different sizes before and after heparin coating. Solid bars represent uncoated PE particles, and dotted bars represent heparin-coated PE particles (HS-PE). (**D**) Flow cytometry analysis of vWF binding to heparin-coated PE particles of different sizes. (**E**) Quantification of surface-area-normalized vWF binding, reflecting relative heparin presentation on heparin-coated PE particles of different sizes. (**F**) Representative microscopy images of non-coated (upper) and heparin-coated (lower) PE-particls (red) bound to vWF-coated surface at the specified shear stress. Scale bar =10 µm. (**G**) Binding of particles to vWF over time and increasing shear stress. Color gradients indicate shear levels (lighter = lower stress, darker = higher stress). * $p \leq 0.05$ Student's t test.

Figure S3, related to Figure 3

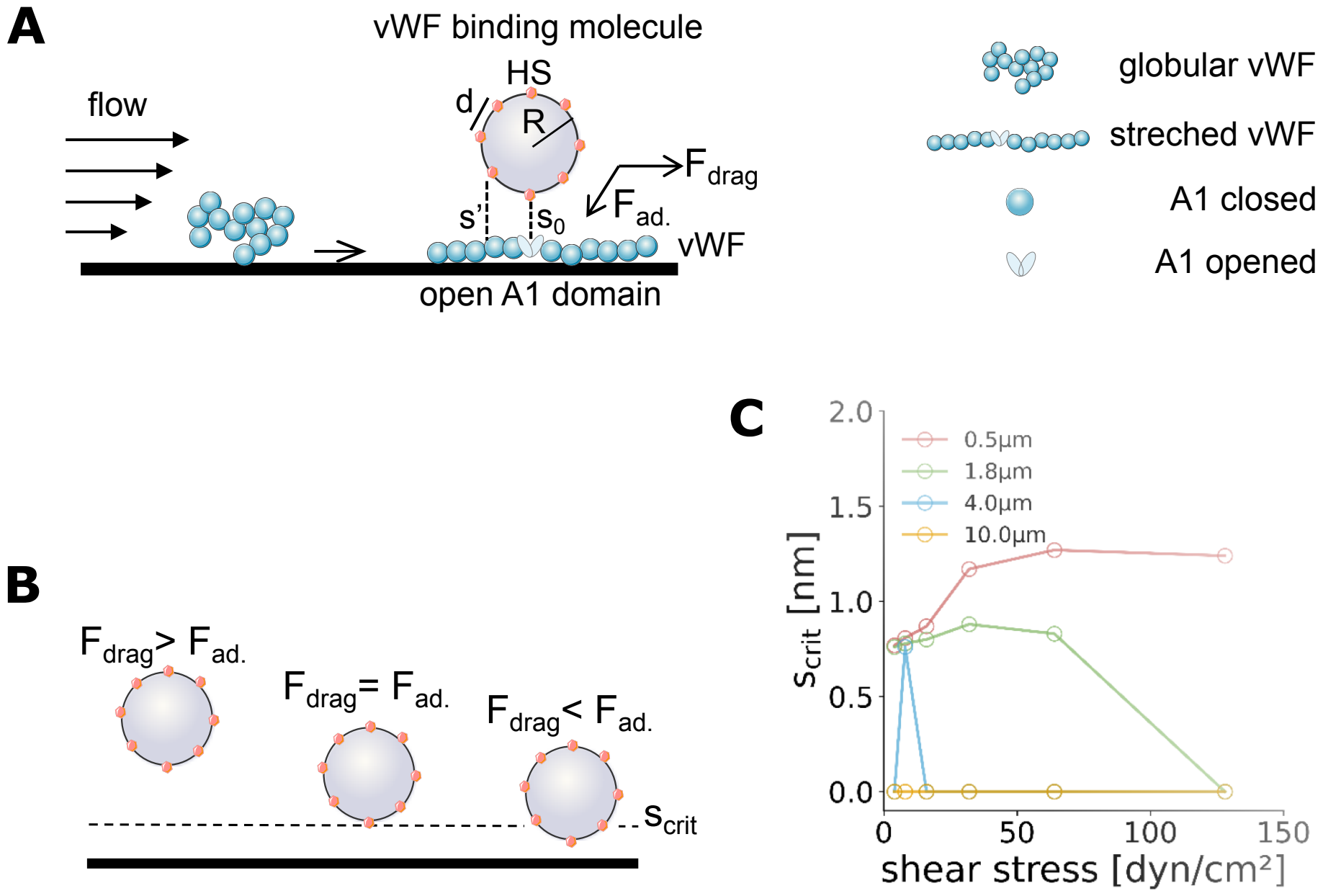

Supplement Figure 3. Binding of blood flowing objects to vWF under shear stress. (**A**) Schematic drawing illustrating the interaction between HS-coated particles and vWF under flow. The drag force (F_{drag}) acting on a particle is determined by Stokes' law, where R is the particle radius, s_0 and s' are the separations between the particle and the surface at different positions. The adhesion force (F_{ad}) is a result of the interaction between vWF and HS. (**B**) Schematic of how the balance between F_{drag} and F_{ad} promote particle adhesion. At a separation above s_{crit}, F_{drag} exceeds F_{ad} and particles detach. At a separation below scrit, F_{drag} is smaller than F_{ad} and particles bind. (**C**) Shown is the change of s_{crit} as a function of the applied shear stress and for particles with different diameters (0.5μm, 1.8μm, 4.0μm, 10.0μm).

Figure S4, related to Figure 3

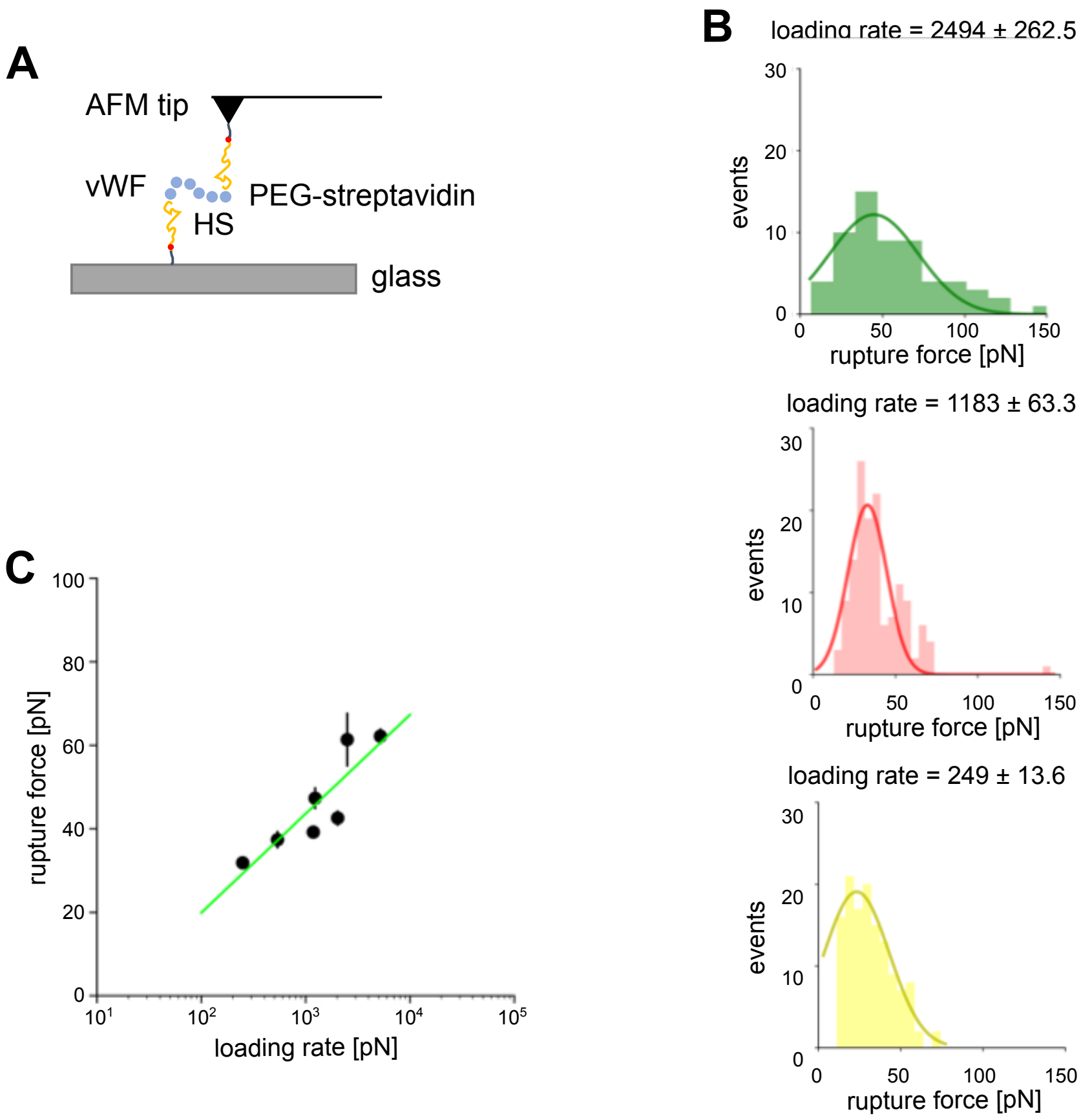

Supplement Figure 4. Analysis of the HS-vWF interaction by single molecule force spectroscopy. (**A**) Schematic of the single-molecule force spectroscopy experiment. Biotinylated HS was attached to the tip of the atomic force microscope and the glass substrate through polyethylene (PEG)-linker immobilized streptavidin. Recombinant vWF was added to the glass slide shortly prior to the measurement. (**B**) Distribution of rupture forces at indicated loading rates. The histograms illustrate the probability distribution of rupture forces required to separate HS from vWF. (C) Relationship between rupture force and loading rate.

Figure S5, related to Figure 5

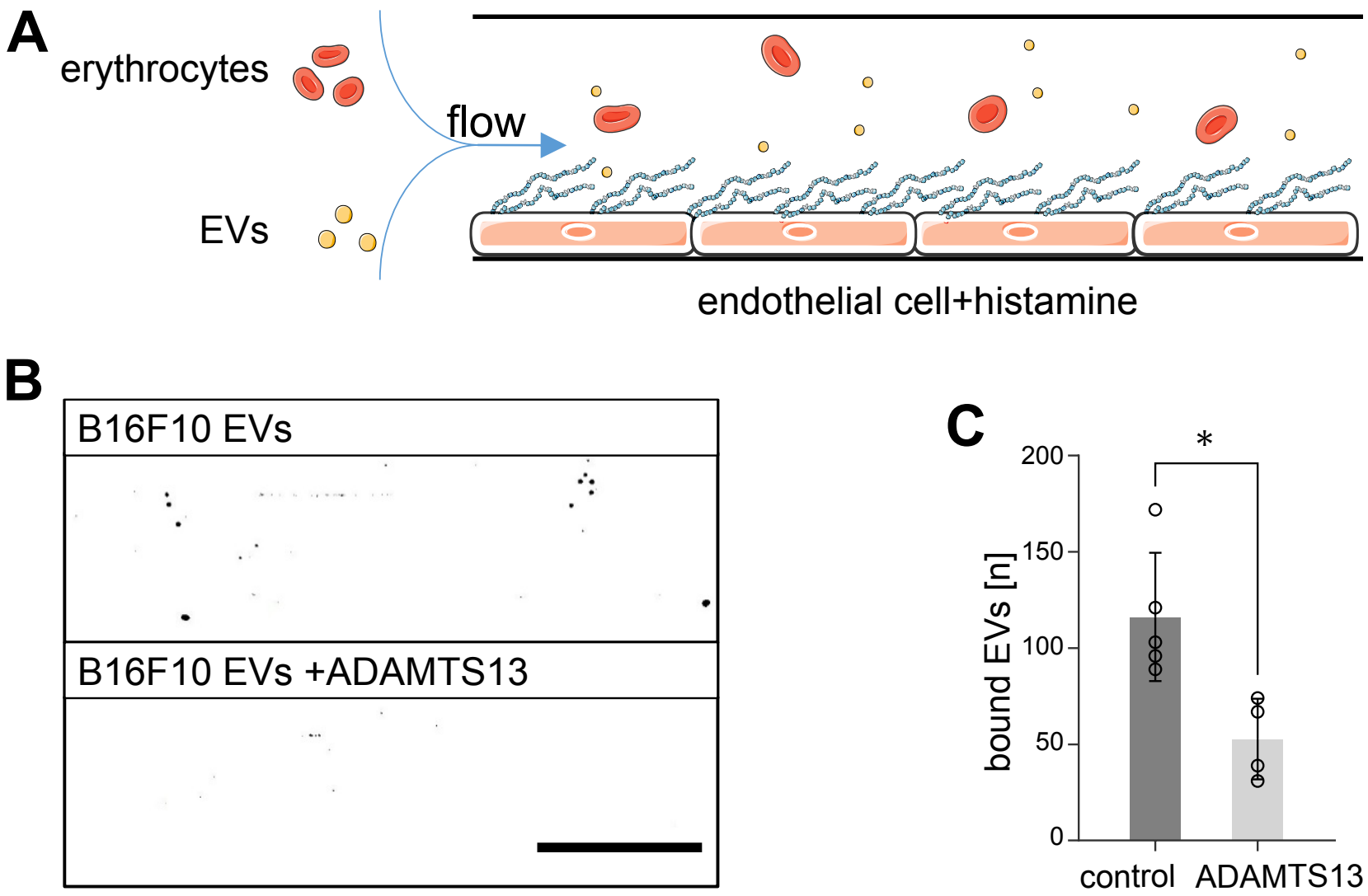

Supplement Figure 5. Binding of EVs to vWF in the absence of platelets. (**A**) Schematic of the microfluidic experiment. EVs were perfused through channels coated with recombinant vWF and erythrocytes. (**B**) Representative images of EV binding to vWF with or without ADAMTS13 treatment. (**C**) Quantification of EV binding to vWF (n=4). * $p \leq 0.05$ Student's *t* test.

Figure S6, related to Figure 6

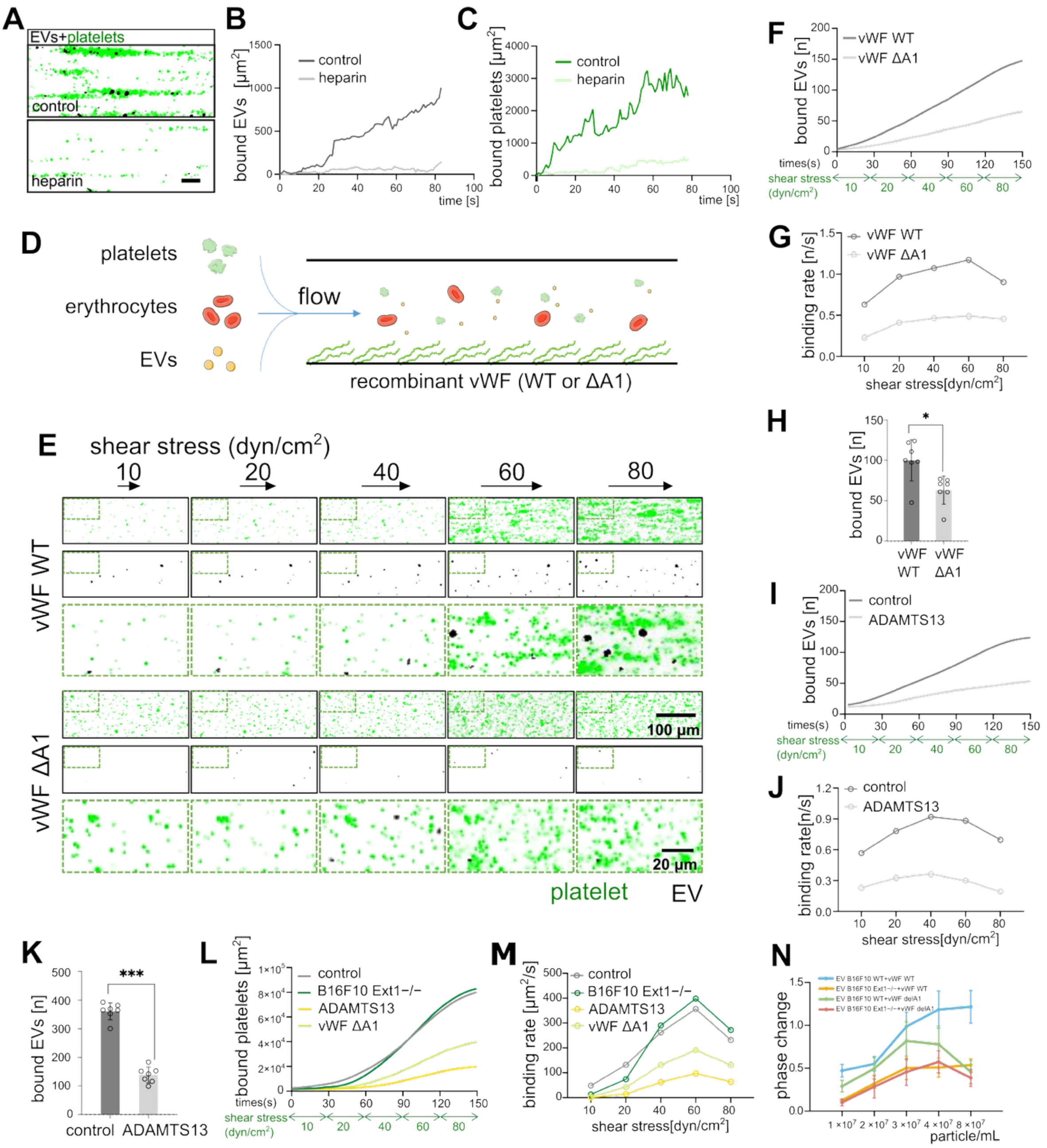

Supplement Figure 6. Binding of melanoma cell-derived EVs to stretched vWF mutants. (**A**) EVs (black) and platelets (green) bound to the vWF with or without heparin inhibition. Scale bars = 20µm (**B**) Binding of EVs and platelets (**C**) over time with or without heparin inhibition. (**D**) Schematic of the microfluidic experiment: EVs were perfused through channels coated with recombinant WT or ΔA1 vWF in the presence of platelets and erythrocytes. (**E**) Time-lapse dual-color fluorescence images of the interaction of platelets (green) and EVs (black) with either WT vWF or ΔA1 vWF under different shear stress conditions. Merged images highlight regions of interest (dashed boxes). (**F**) Binding of EVs to WT or ΔA1 vWF over time and increasing shear stress. (**G**) Binding rate of EVs to WT or ΔA1 vWF as a funtion of shear stress. (**H**) Number of bound EVs at the end of the experiment (n=7). (**I**) Binding of EVs to WT vWF in the presence or absence of ADAMTS13 over time and increasing shear stress. (**J**) Binding rate of EVs to WT vWF in the presence or absence of ADAMTS13 as a funtion of shear stress. (**K**) Number of bound EVs at the end of the experiment (n=7). Data are presented as the mean ± SD, *** *p* ≤ 0.001 Student's *t* test. (**L**) Comparative analysis of the binding of platelets to vWF over time and increasing shear stress. (**M**) Binding rate of platelets to vWF as a function of shear stress. Generated from data shown in (I). (**N**) Phase change after the addition of B16F10 WT or Ext1−/− EVs to WT or Δ

A1 vWF, reflecting binding kinetics and interaction strength measured by surface acoustic wave (SAW) analysis. (n=3) Data are presented as he mean ± SD, * $p \leq 0.05$, *** $p \leq 0.001$ Student's *t* test.

Figure S7, related to Figure 7

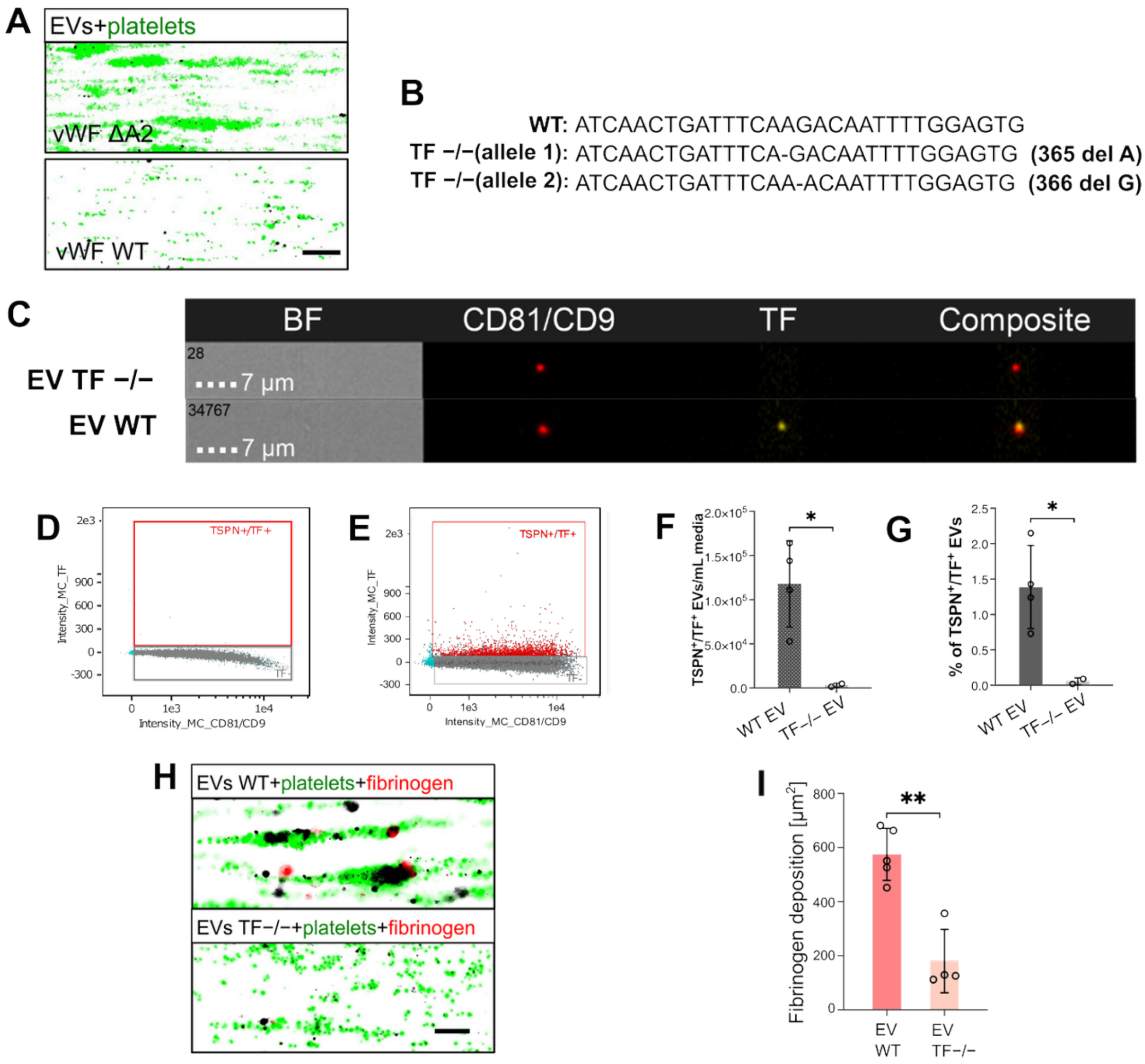

Supplement Figure 7. TF-EV promotes fibrinogen deposition on vWF-bound EV–platelet aggregates under flow. (**A**) EVs (black) and platelets (green) bound to the vWF lacking the A2 domain or wild type vWF. Scale bars = 100µm. **(B)** CRISPR/Cas9 TF knockout in B16F10 cells. Sanger alignment to WT shows 1-bp deletions in both alleles: 365delA (allele 1) and 366delG (allele 2). (**C**) Representative IFCM images of EVs derived from TF-knockout and wild type B16F10 cells, for characterization of CD81/CD9 tetraspanins ("TSPN", as EV markers) and TF. TF+ signals were analyzed within the CD81/CD9-positive EV population. Brightfield (BF), CD81/CD9, TF, and composite images are shown. Scale bars = 7 µm. (**D, E**) Representative dot plots of gated non-swarmed $TSPN^+/TF^+$ EVs based on CD81/CD9 and TF fluorescence intensity in TF−/− EVs and WT EVs. (**F**) Absolute concentration of $TSPN^+/TF^+$ EVs per milliliter of conditioned medium. WT EVs showed a significantly higher abundance of $TSPN^+/TF^+$ EVs compared with TF−/−EVs. (**G**) Relative values of $TSPN^+/TF^+$ EVs among total EVs in WT and TF−/−EV preparations, where the percentages were also signalificantly higher in WT samples. (**H**) Representative fluorescence images showing fibrin(ogen) (red) deposition in the presence of WT EVs or TF−/− EVs together with platelets (green) under flow. Scale bar = 20µm. (**I**) Quantification of EV-induced fibrinogen deposition at the end of the experiment (n=4). * $p \leq 0.05$, Student's *t* test.

Figure S8, related to Figure 8

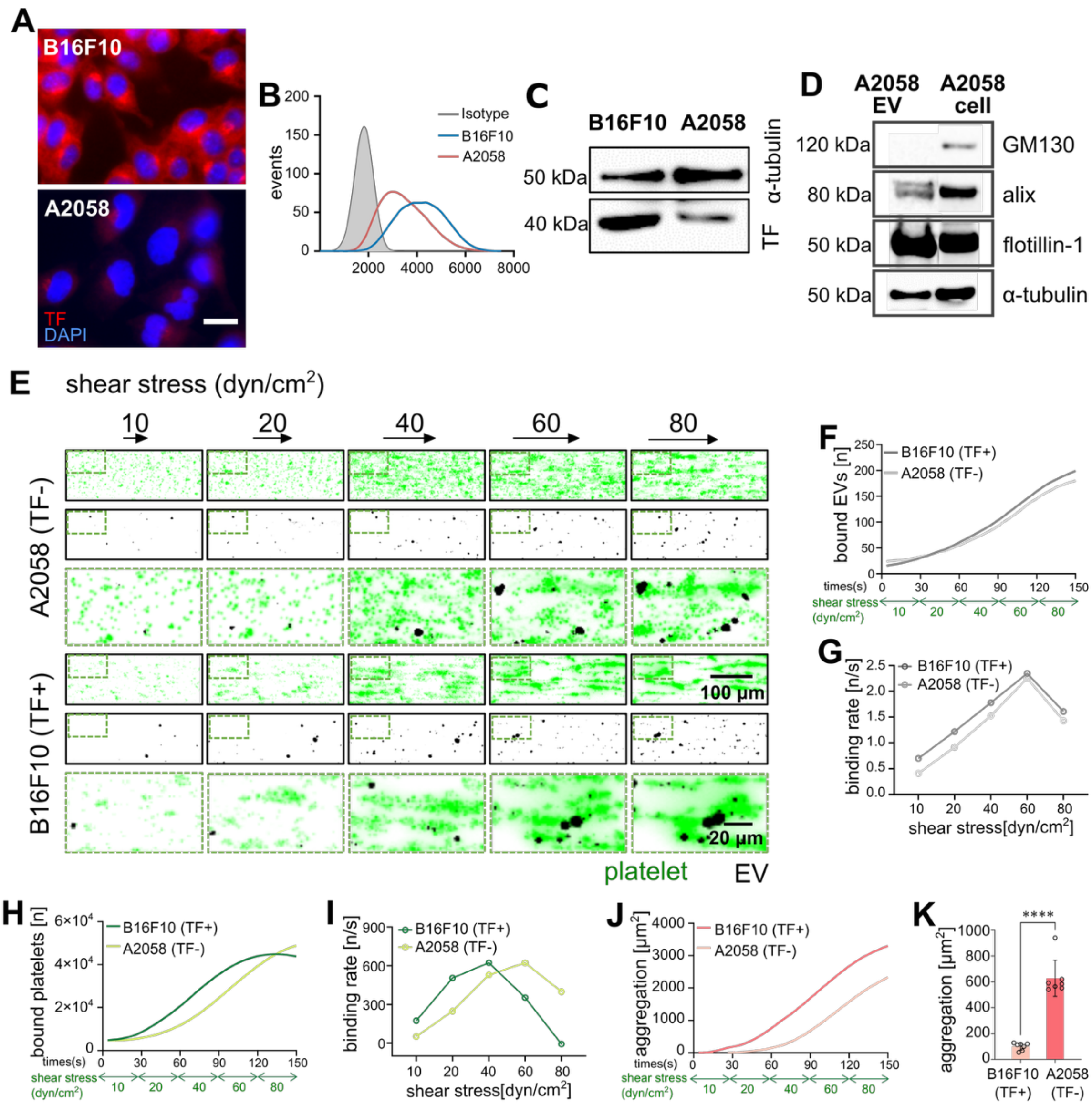

Supplement Figure 8. Binding of EVs of different TF-expressing cell lines to vWF. (**A**) Immunofluorescence staining of TF (red) on B16F10 and A2058 melanoma cells. Nuclei are stained by DAPI (blue). Scale bars = 20μm (**B**) Quantification of TF expression on B16F10 and A2058 cells by flow cytometry and (**C**) by Western blot. α-Tubulin served as a loading control. (**D**) Western blot analysis of EV isolated from A2058 cells. CD81 (22 kDa), flotillin-1 (48 kDa) and alix (96 kDa) were detected in the EVs and cell lysate. The Golgi resident protein GM130 served as a non-EV marker, α-Tubulin served as a loading control. (**E**) Binding of platelets (green) and EVs (black) isolated from A2058 or B16F10 cells with vWF at the indicated shear stresses. Merged images highlight regions of interest (dashed boxes). (**F**) Binding of EVs to vWF coated surfaces over time and increasing shear stress. (**G**) Binding rate of EVs to vWF-coated surfaces as a funtion of shear stress. (**H**) Binding of platelets to vWF coated surfaces in the presence of EVs isolated from B16F10 or A2058 cells over time and increasing shear stress. (**I**) Binding rate of platelets to vWF-coated surfaces as a funtion of shear stress. (**J**) Platelets aggregation in the presence of EVs over time and increasing shear stress. (**K**) Quantification of EV induced platelet aggregation at the end of the flow experiment (n=7). Data are presented as the mean ± SD, **** $p \leq 0.0001$ Student's *t* test.

Figure S9, related to Figure 8

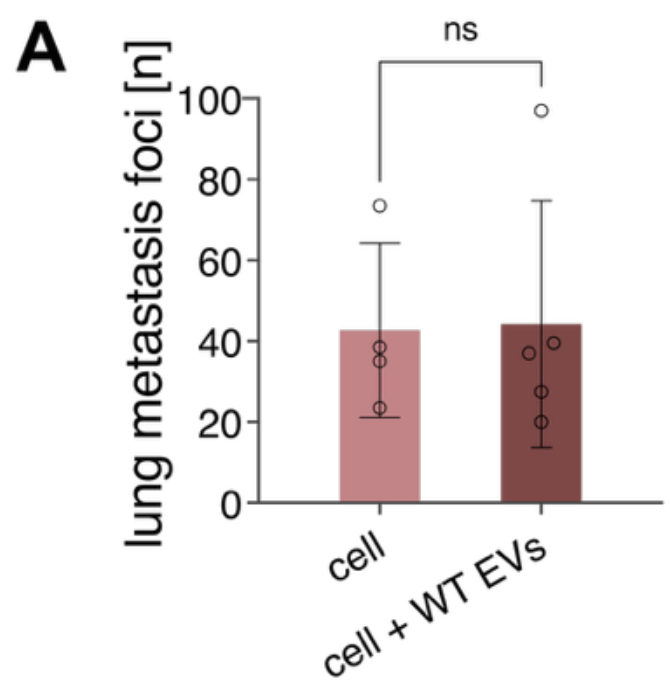

Supplement Figure 9. EV promoted lung metastasis in ADAMTS13−/− mice. (A) In ADAMTS13−/− mice, lung metastatic foci counts are comparable between cells alone and cells + WT EVs. (n = 4-5).

Supplementary Videos

Video S1, related to Figure 1

Platelet binding to vWF fibres under flow. Platelets (green) exhibited increased binding to vWF-coated channel (lower) as shear stress increased, forming a "pearl-on-string" pattern. In contrast, platelet binding to BSA-coated surface (upper) remained consistently low across all applied shear stresses. Scale bar = 100 µm.

Video S2, related to Figure 7

Co-binding of EVs and platelets enhances platelet activation under flow. Platelets (green) and EVs (white) from shCTL (upper) and shTF (lower) MV3 cells bound to vWF at the indicated shear stresses. Activated platelets were marked by CD62P (red). In the upper channel, co-binding of EVs with high TF expression (shCTL, upper) led to increased platelet activation. Scale bar = 100 µm.

References

1 Wang, Y. *et al.* Heparan sulfate dependent binding of plasmatic von Willebrand factor to blood circulating melanoma cells attenuates metastasis. *Matrix Biol* **111**, 76-94 (2022). https://doi.org/10.1016/j.matbio.2022.06.002

2 Merkel, R., Nassoy, P., Leung, A., Ritchie, K. & Evans, E. Energy landscapes of receptor-ligand bonds explored with dynamic force spectroscopy. *Nature* **397**, 50-53 (1999). https://doi.org/10.1038/16219

3 Gockel, L. M. *et al.* Inhibition of Tumor-Host Cell Interactions Using Synthetic Heparin Mimetics. *ACS Appl Mater Interfaces* **13**, 7080-7093 (2021). https://doi.org/10.1021/acsami.0c20744

4 Brenna, S. *et al.* Characterization of brain-derived extracellular vesicles reveals changes in cellular origin after stroke and enrichment of the prion protein with a potential role in cellular uptake. *J Extracell Vesicles* **9**, 1809065 (2020). https://doi.org/10.1080/20013078.2020.1809065

5 Lajqi, T. *et al.* Gut Microbiota-Derived Small Extracellular Vesicles Endorse Memory-like Inflammatory Responses in Murine Neutrophils. *Biomedicines* **10** (2022). https://doi.org/10.3390/biomedicines10020442

6 Hühn, J. *et al.* Selected Standard Protocols for the Synthesis, Phase Transfer, and Characterization of Inorganic Colloidal Nanoparticles. *Chemistry of Materials* **29**, 399-461 (2017). https://doi.org/10.1021/acs.chemmater.6b04738

7 Jiang, Y., Fu, H., Springer, T. A. & Wong, W. P. Electrostatic Steering Enables Flow-Activated Von Willebrand Factor to Bind Platelet Glycoprotein, Revealed by Single-Molecule Stretching and Imaging. *J Mol Biol* **431**, 1380-1396 (2019). https://doi.org/10.1016/j.jmb.2019.02.014

8 Merkel, R., Nassoy, P., Leung, A., Ritchie, K. & Evans, E. Energy landscapes of receptor–ligand bonds explored with dynamic force spectroscopy. *Nature* **397**, 50-53 (1999). https://doi.org/10.1038/16219

9 Arya, M. *et al.* Dynamic force spectroscopy of glycoprotein Ib-IX and von Willebrand factor. *Biophys J* **88**, 4391-4401 (2005). https://doi.org/10.1529/biophysj.104.046318

10 Sacks, D. *et al.* Multisociety Consensus Quality Improvement Revised Consensus Statement for Endovascular Therapy of Acute Ischemic Stroke. *Int J Stroke* **13**, 612-632 (2018). https://doi.org/10.1177/1747493018778713

11 Müller, K., Fedosov, D. A. & Gompper, G. Margination of micro- and nano-particles in blood flow and its effect on drug delivery. *Sci Rep* **4**, 4871 (2014). https://doi.org/10.1038/srep04871

12 Cooley, M. *et al.* Influence of particle size and shape on their margination and wall-adhesion: implications in drug delivery vehicle design across nano-to-micro scale. *Nanoscale* **10**, 15350-15364 (2018). https://doi.org/10.1039/c8nr04042g
